# Complex dynamics of induced vortex formation and thermal-fluid coupling in tri-hybrid nanofluid under localized magnetic field: a novel study

**DOI:** 10.1038/s41598-023-48386-w

**Published:** 2023-11-30

**Authors:** Shabbir Ahmad, Kashif Ali, Humberto Garcia Castellanos, Yashar Aryanfar, Farhan Lafta Rashid, Ahmed S. Hendy, Ahmed Deifalla, Adham E. Ragab, Muhammad Khan, Heba Ghareeb Gomaa

**Affiliations:** 1 National Key Laboratory of Petroleum Resources and Engineering, China University of Petroleum, Beijing, 102249 People’s Republic of China; 2 Department of Basic Sciences and Humanities, Muhammad Nawaz Sharif University of Engineering and Technology, Multan, 60000 Pakistan; 3 Engineering Sciences, Tecnológico Nacional de México IT Ciudad Juárez, Juárez, Chihuahua Mexico; 4 State Key Laboratory of Hydrology-Water Recourses and Hydraulic Engineering, College of Mechanics and Materials, Hohai University, Nanjing, 210098 Jiangsu China; 5 Department of Electric Engineering and Computation, Autonomous University of Ciudad Juárez, Av. Del Charro 450 Norte. Col. Partido Romero, Juárez, Chihuahua México; 6 Petroleum Engineering Department, College of Engineering, University of Kerbala, Karbala, 56001 Iraq; 7 Department of Computational Mathematics and Computer Science, Institute of Natural Sciences and Mathematics, Ural Federal University, 19 Mira St., Yekaterinburg, Russia 620002; 8 Future University in Egypt, New Cairo, 11835 Egypt; 9 Department of Industrial Engineering, College of Engineering, King Saud University, P.O. Box 800, 11421 Riyadh, Saudi Arabia; 10 Department of Materials Science and Engineering, Northwestern Polytechnical University, Xi’an, 710072 Shaanxi People’s Republic of China; 11 Department of Mathematics and Statistics, Institute for Management Information Systems, Suez, Egypt

**Keywords:** Engineering, Mathematics and computing, Nanoscience and technology, Physics

## Abstract

Hybrid nanofluids offer higher stability, synergistic effects, and better heat transfer compared to simple nanofluids. Their higher thermal conductivity, lower viscosity, and interaction with magnetic fields make them ideal for various applications, including materials science, transportation, medical technology, energy, and fundamental physics. The governing partial differential equations are numerically solved by employing a finite volume approach, and the effects of various parameters on the nanofluid flow and thermal characteristics are systematically examined from the simulations based on a self-developed MATLAB code. The parameters included magnetic field strength, the Reynolds number, the nanoparticle volume fraction, and the number and position of the strips in which the magnetic field is localized. It has been noted that the magnetized field induces the spinning of the tri-hybrid nanoparticles, which generates the intricate structure of vortices in the flow. The local skin friction (*CfRe*) and the Nusselt number (Nu) increase significantly when the magnetic field is intensified. Moreover, adding more nanoparticles in the flow enhances both *Nu* and *CfRe*, but with different effects for different nanoparticles. Silver (Ag) shows the highest increase in both *Nu* (52%) and *CfRe* (110%), indicating strong thermal-fluid coupling. Alumina (Al_2_O_3_) and Titanium Dioxide (TiO_2_) show lower increases in both *Nu* (43% and 34%) and *CfRe* (14% and 10%), indicating weaker coupling in the flow. Finally, compared with the localized one, the uniform magnetic field has a minor effect on the flow and temperature distributions.

## Introduction

A lid-driven cavity serves as a representation of flow, in a confined space, where one of the walls moves at a consistent speed. The flow pattern and pressure distribution within the cavity are influenced by the Reynolds number, which measures the relationship between forces and viscous forces without any units. A localized magnetic field refers to a field with intensity in a small area that diminishes rapidly beyond that region. A localized magnetic field can be generated through means such as using magnets, electromagnets, or coils. The impact of a localized field on the flow in a driven cavity is an intriguing research topic with potential applications in biomagnetic fluid dynamics, nanofluid heat transfer, and thermal management of electronic devices. This magnetic field can induce Lorentz forces and Kelvin forces on the fluid thereby modifying both the flow pattern and heat transfer characteristics. The Lorentz force is directly proportional to the conductivity of the fluid while the Kelvin force relates to its susceptibility. The magnitude and direction of these forces rely on factors including the position and orientation of the source as well as properties specific, to each fluid. Banerjee et al.^1^ modeled the blood as a homogeneous Newtonian continuum that exhibits both magnetization and electrical conductivity. The study used the SIMPLER algorithm and the finite volume method to solve the coupled nonlinear system of partial differential equations. The results showed that the stream function plots indicated the field-fluid interactions in laminar flow regimes.

In another study, Abdullah et al.^2^ examined the biomagnetic fluid flow in a lid-driven cavity under the effect of a spatially varying magnetic field. The biomagnetic fluid was modeled as non-conducting and Newtonian. The results showed that the finite element method had stability issues due to extremely steep magnetic field gradients, while the finite difference method had no issues. Chamkha et al.^3^ also investigated mixed convection within a trapezoidal cavity filled with a Cu-water nanofluid under the influence of a continuous magnetic field. The results showed that the magnetic field had a significant effect on both flow and thermal fields, especially for higher Richardson numbers and inclination angles. Nayak et al.^4^ studied the effects of DDNC on hybrid nanofluid flow, heat, and mass transfer in a C-shaped enclosure with two wavy baffles. Results showed that streamlines, velocities, heat/mass transfer rates, and local Nusselt increased with wavy baffle amplitude and other parameters. Average entropy and Bejan number had opposite trends at low and high Ra. Sarangi et al.^5^ compared heat transfer and flow behavior of ternary composite, hybrid, and mono nanofluids with second-order slip conditions. The ternary composite had water, Al_2_O_3_, Graphene, and MWCNT nanoparticles. The results showed that ternary composite nanofluid improved heat transfer and reduced surface viscous drag and motion compared to other nanofluids. Nayak et al.^6^ studied magnetized GO and Al_2_O_3_ nanomaterials’ stagnation point flow around a thin needle in a porous medium. The results showed that increasing the porous matrix reduced surface viscous drag and heat transfer rate for both nanofluids, with GO-water nanofluid having more controlled motion than Al_2_O_3_-water nanofluid as volume fraction increased.

A tri-hybrid nanofluid is a type of nanofluid that contains three different kinds of nanoparticles suspended in a base fluid. Nanofluids are fluids that have enhanced thermal properties due to the addition of nanoparticles. Tri-hybrid nanofluids are expected to have even higher thermal conductivity and heat transfer performance than single or hybrid nanofluids. Rauf et al.^7^ analyzed the magnetohydrodynamic flow and heat transfer of a micropolar tri-hybrid nanofluid between porous surfaces in a rotating system. They considered thermal radiation and Hall current effects and used the finite volume method for numerical solutions. The results showed that tri-hybrid nanofluids had superior heat transfer rates compared to hybrid nanofluids and nanofluids. Similarly, Arif et al.^8^ explored the thermal performance of a tri-hybrid nanofluid with spherical, platelet, and cylindrical nanoparticles in engine oil on an infinite vertical plate in a rotating frame. The results showed that tri-hybrid nanofluid had enhanced thermal characteristics compared to engine oil. On the other hand, Sohail et al.^9^ studied the use of tri-hybrid nanoparticles in a Prandtl fluid model over a heated stretched sheet. They modeled the problem with a generalized heat flux model and heat generation/absorption and used the finite element algorithm for numerical solutions. The results showed that tri-hybrid nanoparticles increased the fluid velocity compared to pure fluid, nanofluid, and hybrid nanomaterial. Ali and Kai et al.^10–15^ have made notable contributions to the investigation of the thermal properties of various nanofluids in intriguing problem domains.

Animasaun et al.^16^ analyzed the Darcy flow of ternary-hybrid nanofluid and its relation to leading-edge accretion. The results showed that velocity decreased significantly with leading-edge accretion, while other parameters had minor effects. Similarly, Arif et al.^17^ explored the heat transfer of a tri-hybrid nanofluid with spherical, platelet, and cylindrical nanoparticles in blood for pharmaceutical applications. The Atangana–Baleanu time-fractional operator and the Laplace and Fourier transforms for numerical solutions were used. The results showed that tri-hybrid nanofluid had enhanced thermal characteristics compared to blood. On the other hand, Animasaun et al.^18^ studied the constant-pressure boundary-layer flow with a large Grashof number. The results showed that velocity was minimal with a stationary surface and temperature distribution was minimal with a moving one. Lower skin friction occurred with higher nanoparticle volume. Furthermore, Saranya et al.^19^ investigated quartic-type homogeneous-heterogeneous reactions in a tri-hybrid nanofluid on a stationary/moving flat plate. Tiwari-Das model and the finite element algorithm for numerical solutions were used. The results showed that homogeneous and heterogeneous reactions, catalyst diffusivity, and Schmidt number affected the concentration fields. Rajput et al.^20^ analyzed the flow of THN (graphene-graphene oxide-silver/water) between squeezing parallel plates, relevant to industrial applications. The results showed that squeezing and magnetic parameters reduced nanofluid velocity, while nanoparticle concentration, Prandtl number, and radiation effect-controlled temperature.

Concentration had overshoot with higher temperature gradients, and local Nusselt and Sherwood numbers varied by nanofluid type and other parameters. Similarly, Bilal et al.^21^ explored the magnetic dipole and heat transfer of a ternary hybrid Carreau Yasuda nano liquid over a vertical stretching sheet. They combined Al_2_O_3_, SiO_2_, and TiO_2_ nanoparticles in a Carreau Yasuda fluid to create tri-hybrid nanofluids. The parametric continuation method for numerical solutions has been used and considered heat source/sink and Darcy Forchheimer effects. The results showed that adding nanoparticles improved energy and momentum profiles in engine oil, with Thnf having superior thermal energy transfer. Ferrohydrodynamic interaction decreased fluid velocity, while nanoparticle inclusion increased it. Latha et al.^22^ analyzed the magnetohydrodynamic non-Newtonian flow and heat transfer of a tri-hybrid nanofluid (EO doped with graphene, gold, and cobalt oxide nanoparticles) in Hiemenz plane stagnation flow. The results showed that velocity decreased with ternary nanofluid and magnetic parameters, while temperature increased with thermal radiation. Similarly, Bilal et al.^23^ explored the 3D permeable surface with dual stretching. The results showed that velocity, temperature, and concentration were higher for ternary group 1 with lower-density particles than for group 2 with denser particles. Temperature and concentration distribution were affected by Soret and Dufour numbers, and skin friction was higher for the group with less dense particles. More details about this topic are available in the papers by Rashid and Ayub et al.^24–30^.

A tri-hybrid nanofluid comprises nanoparticles of Ag, Al_2_O_3_ and TiO_2_. These nanoparticles possess chemical, optical, and electrical properties that depend on their nature and synthesis. Ag nanoparticles are particles known for their electrical conductivity and strong plasmonic effect. They can. Scatter light, while also exhibiting antibacterial and antifungal properties^31^. On the other hand, Al_2_O_3_ nanoparticles are particles with high thermal stability but low electrical conductivity. They exist in crystalline phases like alpha, gamma, delta, and theta. Among these phases, alpha Al_2_O_3_ is the most stable and widely used due to its structure and remarkable hardness. It finds applications as abrasives, catalysts, coatings, fillers, and additives^32^. Lastly, TiO_2_ nanoparticles are semiconducting particles with a band gap and high refractive index. They have three crystalline phases; anatase, rutile, and brookite. Anatase and rutile are the phases with tetragonal structures but differ in lattice parameters and symmetry. These two phases find applications in areas such, as photocatalysis, pigmentation, sensors, solar cells, and biomedical materials^33,34^.

The Alternating Direction Implicit (ADI) technique is an approach used to solve PDEs in dimensions. It simplifies these equations by breaking them down into one-dimensional PDEs. It can be applied to both parabolic PDEs, including the Poisson, heat, and diffusion equations. One of the benefits of using the ADI method is its ability to achieve accuracy and stability while keeping computational costs low. To effectively use the ADI method certain parameters, need to be considered, such as the time step size and shift parameters. These parameters play a role, in controlling the iteration process and achieving results based on the specific properties of the PDE being solved. Compared to methods the ADI technique has many advantages. It helps in reducing complexity by avoiding solving sparse linear systems. Additionally, it handles uniform grids and non-constant coefficients with ease while preventing numerical instabilities from occurring. Furthermore, it can be extended to dimensions and complex PDEs without much difficulty. Some guidelines and formulas for selecting these parameters are available in the literature^35–39^. Overall utilizing the ADI method provides advantages, for solving PDEs in multiple dimensions while maintaining accuracy and stability.

This study aims to fill the knowledge gaps on how nanofluids with three types of particles affect vortex formation in lid-driven cavities, and it presents the objectives and outcomes of this research. Additionally, it investigates how localized magnetic fields influence the flow and heat transfer properties within these cavities. To solve the equations related to this problem a method based on the ADI technique is developed. Furthermore, a comprehensive analysis is conducted to explore parameters affecting vortex generation and heat transfer enhancement in the nanofluids. Lastly, the study investigates how vortex formation affects flow patterns, temperature distribution, Nusselt number, and skin friction.

The primary research questions which we try to answer in this paper may be presented as follows:

- How the magnetic field, structured as vertical and horizontal strips, affect the main vortex within the flow?
- What is the way the magnetic number, the Reynolds number, and the nanostructure of the fluid collectively influence the skin friction factor and the Nusselt number?
- How does increasing the width of the magnetic field (characterized by the parameter (*L*) influence the average Nusselt number (*Nu*) and skin friction factor (*CfRe*)?
- How does the increasing nanoparticle volume fraction influence the average Nusselt number, and what advantages do tri-hybrid nanofluids, comprising Ag, Al_2_O_3_, and TiO_2_ nanoparticles, offer in terms of enhancing heat transfer rates?
- How do the way the parameters A1 and A2 (controlling the strength of the magnetic field) influence the average Nusselt number?

## Problem statement

**Figure 1:**
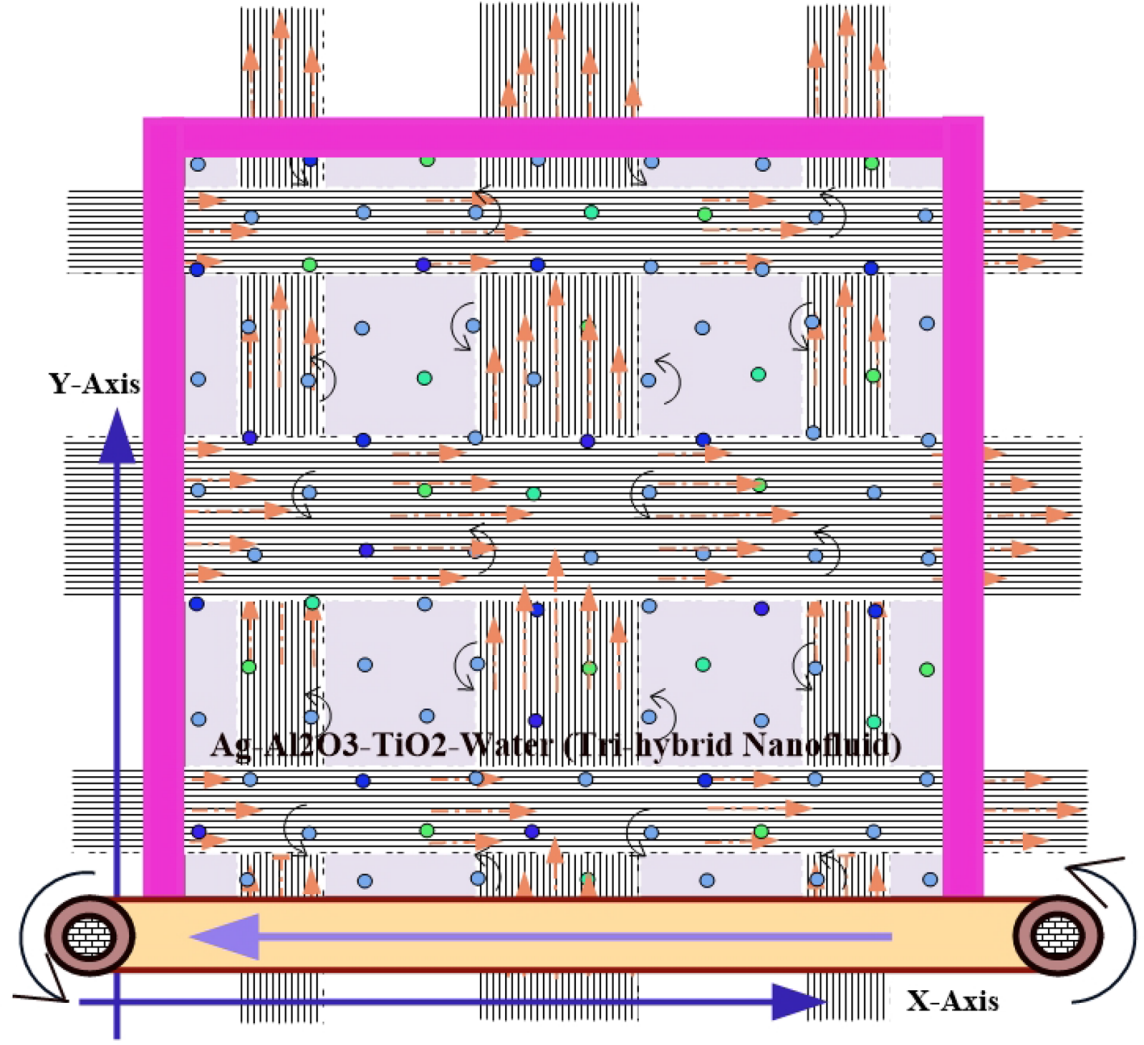
A physical diagram of a square cavity with a moving lid and a localized magnetic field. The straight arrows indicate the region where the magnetic field is applied.

Figure 1 illustrates a two-dimensional square cavity of length L with an aspect ratio of one unit. The cavity contains a mixture of Ag-Al_2_O_3_-TiO_2_ nanoparticles and water. The solid particles have the same shape and size, and their diameter is 100 nm. The lower horizontal wall has a higher temperature (T_h_) than the upper one (T_c_), which creates the buoyancy effect. The lower horizontal wall also moves from right to left with a uniform velocity. Both vertical walls are insulated. The nanoparticles and the fluid phase have the same temperature. The density is the only property that varies for the nanoparticles and the fluid. Table 3 shows the thermos-physical properties of water and Ag–Al_2_O_3_–TiO_2_ at the reference temperature. A continuous magnetic field is applied to the bottom wall of the enclosure. To model the evolution of fluid flow and heat transfer within the cavity, we use a single-phase model (SPM). This model provides the governing equations for mass, momentum, and energy conservation in a two-dimensional Cartesian coordinate system.

### Basic assumptions

The following assumptions are the basis of this study:

A magnetized source generates a magnetic field with the strength H defined by the equation:

$$ \begin{aligned} \tilde{H}_{1} (x,y) & = H_{0} \left\{ {\tanh A^{\prime}_{1} \left( {x - x_{1} } \right) - \tanh A^{\prime}_{2} \left( {x - x_{2} } \right)} \right\},\quad \tilde{H}_{2} (x,y) = H_{0} \left\{ {\tanh A^{\prime}_{1} \left( {x - x_{3} } \right) - \tanh A^{\prime}_{2} \left( {x - x_{4} } \right)} \right\} \\ \tilde{H}_{3} (x,y) & = H_{0} \left\{ {\tanh A^{\prime}_{1} \left( {x - x_{5} } \right) - \tanh A^{\prime}_{2} \left( {x - x_{6} } \right)} \right\}\quad \tilde{H}_{4} (x,y) = H_{0} \left\{ {\tanh A^{\prime}_{1} \left( {y - y_{1} } \right) - \tanh A^{\prime}_{2} \left( {y - y_{2} } \right)} \right\} \\ \tilde{H}_{5} (x,y) & = H_{0} \left\{ {\tanh A^{\prime}_{1} \left( {y - y_{3} } \right) - \tanh A^{\prime}_{2} \left( {y - y_{4} } \right)} \right\},\quad \tilde{H}_{6} (x,y) = H_{0} \left\{ {\tanh A^{\prime}_{1} \left( {y - y_{5} } \right) - \tanh A^{\prime}_{2} \left( {y - y_{6} } \right)} \right\} \\ \end{aligned} $$

in the strips mentioned by $\,\,x_{1} \le x \le x_{2} ,\,\,\,\,x_{3} \le x \le x_{4} ,\,\,\,x_{5} \le x \le x_{6}$; $0 \le y \le L$ and $\,\,y_{1} \le y \le y_{2} ,\,\,\,\,y_{3} \le y \le y_{4} ,\,\,\,\,y_{5} \le y \le y_{6}$; $0 \le x \le L$ respectively. A square cavity is shown in the diagram, with insulated vertical walls and horizontal walls with fixed temperatures. The bottom wall moves steadily to the left, while the top wall is stationary. The cavity is filled with a tri-hybrid nanofluid, which is a mixture of water and solid nanoparticles of Ag, Al_2_O_3_, and TiO_2_. The nanofluid is in thermal equilibrium and has constant properties, including density, thermal conductivity, viscosity, and specific heat. It also behaves like a Newtonian fluid, meaning that it is incompressible and has laminar flow.

## Mathematical representation

The Navier–Stokes Equations (NSE) are derived from these principles for the fluid: It is continuous, has constant density and viscosity, and conserves mass, momentum, and energy.

These equations are written in this dimensional form^40^:

*Continuity equation:*

1 $$ \frac{{\partial \tilde{U}}}{\partial x} + \frac{{\partial \tilde{V}}}{\partial y} = 0, $$

*Momentum equation:*

2 $$ \frac{{\partial \tilde{U}}}{{\partial t^{\prime}}} + \left( {\tilde{V}\frac{{\partial \tilde{U}}}{\partial y} + \tilde{U}\frac{{\partial \tilde{U}}}{\partial x}} \right) = - \frac{1}{{\overset{\lower0.5em\hbox{$\smash{\scriptscriptstyle\frown}$}}{\rho }_{hnf} }}\frac{\partial P}{{\partial x}} + \overset{\lower0.5em\hbox{$\smash{\scriptscriptstyle\frown}$}}{\upsilon }_{hnf} \left( {\frac{{\partial^{2} \tilde{U}}}{{\partial y^{2} }} + \frac{{\partial^{2} \tilde{U}}}{{\partial x^{2} }}} \right) + \frac{{\overline{\mu }_{o} \tilde{M}}}{{\overset{\lower0.5em\hbox{$\smash{\scriptscriptstyle\frown}$}}{\rho }_{hnf} }}\frac{{\partial \tilde{H}}}{\partial x}, $$

3 $$ \frac{{\partial \tilde{V}}}{{\partial t^{\prime}}} + \left( {\tilde{U}\frac{{\partial \tilde{V}}}{\partial x} + \tilde{V}\frac{{\partial \tilde{V}}}{\partial y}} \right) = - \frac{1}{{\overset{\lower0.5em\hbox{$\smash{\scriptscriptstyle\frown}$}}{\rho }_{hnf} }}\frac{\partial P}{{\partial y}} + \overset{\lower0.5em\hbox{$\smash{\scriptscriptstyle\frown}$}}{\upsilon }_{hnf} \left( {\frac{{\partial^{2} \tilde{V}}}{{\partial x^{2} }} + \frac{{\partial^{2} \tilde{V}}}{{\partial y^{2} }}} \right) + \frac{{\overline{\mu }_{o} \tilde{M}}}{{\overset{\lower0.5em\hbox{$\smash{\scriptscriptstyle\frown}$}}{\rho }_{hnf} }}\frac{{\partial \tilde{H}}}{\partial y}, $$

*Energy Equation:*

4 $$ \begin{gathered} \frac{{\left( {\overset{\lower0.5em\hbox{$\smash{\scriptscriptstyle\frown}$}}{\rho } c_{p} } \right)_{hnf} }}{{k_{hnf} }}\left( {\tilde{U}\frac{\partial T}{{\partial x}} + \tilde{V}\frac{\partial T}{{\partial y}}} \right) + \left( {\frac{{\overline{\mu }_{o} }}{{k_{hnf} }}} \right)T\frac{{\partial \tilde{M}}}{\partial T}\left( {\tilde{V}\frac{{\partial \tilde{H}}}{\partial y} + \tilde{U}\frac{{\partial \tilde{H}}}{\partial x}} \right) \hfill \\ = \nabla^{2} T + \left( {\frac{{\tilde{\mu }_{hnf} }}{{k_{hnf} }}} \right)\left\{ {2\left( {\frac{{\partial \tilde{U}}}{\partial x}} \right)^{2} + \left( {\frac{{\partial \tilde{V}}}{\partial x} + \frac{{\partial \tilde{U}}}{\partial y}} \right)^{2} + 2\left( {\frac{{\partial \tilde{V}}}{\partial y}} \right)^{2} } \right\}. \hfill \\ \end{gathered} $$

Eliminating the pressure term results in:

5 $$ \begin{aligned} & \frac{\partial }{{\partial t^{\prime}}}\left( {\frac{{\partial \tilde{U}}}{\partial y} - \frac{{\partial \tilde{V}}}{\partial x}} \right) + \overset{\lower0.5em\hbox{$\smash{\scriptscriptstyle\frown}$}}{V} \frac{\partial }{\partial y}\left( {\frac{{\partial \tilde{U}}}{\partial y} - \frac{{\partial \tilde{V}}}{\partial x}} \right) + \tilde{U}\frac{\partial }{\partial x}\left( {\frac{{\partial \tilde{U}}}{\partial y} - \frac{{\partial \tilde{V}}}{\partial x}} \right) \\ & \quad = \overset{\lower0.5em\hbox{$\smash{\scriptscriptstyle\frown}$}}{\upsilon }_{thnf} \left( {\frac{{\partial^{2} }}{{\partial x^{2} }} + \frac{{\partial^{2} }}{{\partial y^{2} }}} \right)\left( {\frac{{\partial \tilde{U}}}{\partial y} - \frac{{\partial \tilde{V}}}{\partial x}} \right) + \left( {\frac{{\partial \left( {\frac{{\overline{\mu }_{0} \tilde{M}}}{{\overset{\lower0.5em\hbox{$\smash{\scriptscriptstyle\frown}$}}{\rho }_{thnf} }}\frac{{\partial \tilde{H}}}{\partial x}} \right)}}{\partial y} - \frac{{\partial \left( {\frac{{\overline{\mu }_{0} \tilde{M}}}{{\overset{\lower0.5em\hbox{$\smash{\scriptscriptstyle\frown}$}}{\rho }_{thnf} }}\frac{{\partial \tilde{H}}}{\partial y}} \right)}}{\partial x}} \right). \\ \end{aligned} $$

### Boundary conditions

The current obstacle has the following size and boundary conditions:

*Left and Right Vertical Walls (Adiabatic):* The heat flux across these walls is zero, i.e.,

6a $$ \,\tilde{U}(0,\,y) = \tilde{U}(L,\,y) = 0,\,\,\,\,\,\,\,\left( {\frac{\partial T}{{\partial x}}} \right)_{x = 0} = \, \left( {\frac{\partial T}{{\partial x}}} \right)_{x = L} = 0,\,\,\,\,\,\,\tilde{V}(0,\,y) = \tilde{V}(L,\,y) = 0;\,\,\,\,\,\,\,\,\,\,0 < y < L $$

*Upper Horizontal Wall:*

6b $$ \tilde{U}(x,\,L) = 0,\,\,\,\,\,\,\,\,T(x,\,L) = T_{c} \,,\,\,\,\,\, \, \tilde{V}(x,\,L) = 0\,;\,\,\,\,\,\,\,\,\,\,\,\,\,\,\,0 < x < L $$

*Lower Horizontal Wall:*

6c $$ \tilde{U}(x,\,0) = - V_{0} , \, \,\,\,\,T(x,\,0) = T_{h} \,,\,\,\,\,\,\,\,\,\tilde{V}(x,\,0) = 0\,;\,\,\,\,\,\,\,\,\,\,\,\,0 < x < L. $$

### The characteristics of tri hybrid nanofluids (Ag–Al_2_O_3_–TiO_2_)

This section presents the thermophysical properties of the tri-hybrid nanofluids used in this study, which are essential for addressing heat transfer issues. These nanofluids are a mixture of water and Ag–Al_2_O_3_–TiO_2_ nanoparticles. The density, specific heat, thermal conductivity, and viscosity of these tri-hybrid nanofluids were taken from previously published literature sources^41–44^. The description of all the symbols which appear in Table 2 is given below in Table 1, while Table 2 provides a comprehensive overview of the symbols used and the corresponding values for these properties.

**Table 1:** Explanation of the symbols in Table 2.

	For Tri-hybrid nanofluids	For Hybrid nanofluids	For nanofluids	For base fluids (water)	Silver ($s_{1}$)	For solid	
						Aluminium oxide ($s_{2}$)	Titanium dioxide ($s_{3}$)
Thermal conductivity	$k_{thnf}$	$k_{hnf}$	$k_{nf}$	$k_{f}$	$k_{s1}$	$k_{s2}$	$k_{s3}$
Density	$$\overset{\lower0.5em\hbox{$\smash{\scriptscriptstyle\frown}$}}{\rho }_{thnf}$$			$$\overset{\lower0.5em\hbox{$\smash{\scriptscriptstyle\frown}$}}{\rho }_{f}$$	$$\overset{\lower0.5em\hbox{$\smash{\scriptscriptstyle\frown}$}}{\rho }_{s1}$$	$$\overset{\lower0.5em\hbox{$\smash{\scriptscriptstyle\frown}$}}{\rho }_{s2}$$	$$\overset{\lower0.5em\hbox{$\smash{\scriptscriptstyle\frown}$}}{\rho }_{s3}$$
Electrical conductivity	$\sigma_{thnf}$	$\sigma_{hnf}$	$\sigma_{nf}$	$\sigma_{f}$	$\sigma_{s1}$	$\sigma_{s2}$	$\sigma_{s3}$
Viscosity	$\mu_{thnf}$	$\mu_{hnf}$	$\mu_{nf}$	$\mu_{f}$			
Nanoparticles volume fraction					$$\overset{\lower0.5em\hbox{$\smash{\scriptscriptstyle\frown}$}}{\varphi }_{1}$$	$$\overset{\lower0.5em\hbox{$\smash{\scriptscriptstyle\frown}$}}{\varphi }_{2}$$	$$\overset{\lower0.5em\hbox{$\smash{\scriptscriptstyle\frown}$}}{\varphi }_{3}$$

**Table 2:** Thermophysical Properties of Conventional and Tri-hybrid Nanofluids.

Properties	Tri-hybrid nanofluid(thnf)
Density	$$\overset{\lower0.5em\hbox{$\smash{\scriptscriptstyle\frown}$}}{\rho }_{thnf} = \left( {1 - \overset{\lower0.5em\hbox{$\smash{\scriptscriptstyle\frown}$}}{\varphi }_{3} } \right)\left[ {\left( {1 - \overset{\lower0.5em\hbox{$\smash{\scriptscriptstyle\frown}$}}{\varphi }_{1} } \right)\left\{ {\left( {1 - \overset{\lower0.5em\hbox{$\smash{\scriptscriptstyle\frown}$}}{\varphi }_{2} } \right)\overset{\lower0.5em\hbox{$\smash{\scriptscriptstyle\frown}$}}{\rho }_{f} + \overset{\lower0.5em\hbox{$\smash{\scriptscriptstyle\frown}$}}{\varphi }_{1} \overset{\lower0.5em\hbox{$\smash{\scriptscriptstyle\frown}$}}{\rho }_{s1} } \right\} + \overset{\lower0.5em\hbox{$\smash{\scriptscriptstyle\frown}$}}{\varphi }_{2} \overset{\lower0.5em\hbox{$\smash{\scriptscriptstyle\frown}$}}{\rho }_{s2} } \right] + \overset{\lower0.5em\hbox{$\smash{\scriptscriptstyle\frown}$}}{\varphi }_{3} \overset{\lower0.5em\hbox{$\smash{\scriptscriptstyle\frown}$}}{\rho }_{s3}$$
Heat capacity	$$(\overset{\lower0.5em\hbox{$\smash{\scriptscriptstyle\frown}$}}{\rho } cp)_{thnf} = \left( {1 - \overset{\lower0.5em\hbox{$\smash{\scriptscriptstyle\frown}$}}{\varphi }_{3} } \right)\left[ {\left( {1 - \overset{\lower0.5em\hbox{$\smash{\scriptscriptstyle\frown}$}}{\varphi }_{2} } \right)\left\{ {\left( {1 - \overset{\lower0.5em\hbox{$\smash{\scriptscriptstyle\frown}$}}{\varphi }_{1} } \right)\overset{\lower0.5em\hbox{$\smash{\scriptscriptstyle\frown}$}}{\rho }_{f} + \overset{\lower0.5em\hbox{$\smash{\scriptscriptstyle\frown}$}}{\varphi }_{1} \overset{\lower0.5em\hbox{$\smash{\scriptscriptstyle\frown}$}}{\rho }_{s1} } \right\} + \overset{\lower0.5em\hbox{$\smash{\scriptscriptstyle\frown}$}}{\varphi }_{2} \overset{\lower0.5em\hbox{$\smash{\scriptscriptstyle\frown}$}}{\rho }_{s2} } \right] + \overset{\lower0.5em\hbox{$\smash{\scriptscriptstyle\frown}$}}{\varphi }_{3} \overset{\lower0.5em\hbox{$\smash{\scriptscriptstyle\frown}$}}{\rho }_{s3}$$
viscosity	$$\mu_{thnf} = \frac{{\mu_{{_{f} }} }}{{(1 - \overset{\lower0.5em\hbox{$\smash{\scriptscriptstyle\frown}$}}{\varphi }_{1} )^{2.5} (1 - \overset{\lower0.5em\hbox{$\smash{\scriptscriptstyle\frown}$}}{\varphi }_{2} )^{2.5} (1 - \overset{\lower0.5em\hbox{$\smash{\scriptscriptstyle\frown}$}}{\varphi }_{3} )^{2.5} }}$$
Thermal conductivity	$$\frac{{k_{thnf} }}{{k_{hnf} }} = \frac{{k_{s3} + \left( {n - 1} \right)(k_{hnf} - (n - 1)\overset{\lower0.5em\hbox{$\smash{\scriptscriptstyle\frown}$}}{\varphi }_{s3} )(k_{hnf} - k_{s3} )}}{{k_{s3} + \left( {n - 1} \right)k_{hnf} + \overset{\lower0.5em\hbox{$\smash{\scriptscriptstyle\frown}$}}{\varphi }_{s3} (k_{hnf} - k_{s3} )}}$$ where $$\frac{{k_{hnf} }}{{k_{nf} }} = \frac{{k_{s2} - \left( {n - 1} \right)\overset{\lower0.5em\hbox{$\smash{\scriptscriptstyle\frown}$}}{\varphi }_{2} (k_{nf} - k_{s3} ) + \left( {n - 1} \right)k_{nf} }}{{k_{s2} + \overset{\lower0.5em\hbox{$\smash{\scriptscriptstyle\frown}$}}{\varphi }_{s2} (k_{nf} - k_{s3} ) + \left( {n - 1} \right)k_{nf} }}$$ and $$\frac{{k_{nf} }}{{k_{f} }} = \frac{{k_{s1} - \left( {n - 1} \right)\overset{\lower0.5em\hbox{$\smash{\scriptscriptstyle\frown}$}}{\varphi }_{1} (k_{f} - k_{s1} ) + \left( {n - 1} \right)k_{f} }}{{k_{s1} + \overset{\lower0.5em\hbox{$\smash{\scriptscriptstyle\frown}$}}{\varphi }_{1} (k_{f} - k_{s1} ) + \left( {n - 1} \right)k_{f} }}$$
Electric conductivity	$$\frac{{\sigma_{thnf} }}{{\sigma_{nf} }} = \frac{{\sigma_{s3} - 2\overset{\lower0.5em\hbox{$\smash{\scriptscriptstyle\frown}$}}{\varphi }_{3} (\sigma_{nf} - \sigma_{s3} ) + 2\sigma_{nf} }}{{\sigma_{s3} + \overset{\lower0.5em\hbox{$\smash{\scriptscriptstyle\frown}$}}{\varphi }_{3} (\sigma_{nf} - \sigma_{s3} ) + 2\sigma_{nf} }}$$ where $$\frac{{\sigma_{hnf} }}{{\sigma_{nf} }} = \frac{{\sigma_{s2} - 2\overset{\lower0.5em\hbox{$\smash{\scriptscriptstyle\frown}$}}{\varphi }_{2} (\sigma_{nf} - \sigma_{s2} ) + 2\sigma_{nf} }}{{\sigma_{s2} + \overset{\lower0.5em\hbox{$\smash{\scriptscriptstyle\frown}$}}{\varphi }_{2} (\sigma_{nf} - \sigma_{s2} ) + 2\sigma_{nf} }}$$ and $$\frac{{\sigma_{nf} }}{{\sigma_{f} }} = \frac{{\sigma_{s1} - 2\overset{\lower0.5em\hbox{$\smash{\scriptscriptstyle\frown}$}}{\varphi }_{1} (\sigma_{f} - \sigma_{s1} ) + 2\sigma_{f} }}{{\sigma_{s1} + \overset{\lower0.5em\hbox{$\smash{\scriptscriptstyle\frown}$}}{\varphi }_{1} (\sigma_{f} - \sigma_{s1} ) + 2\sigma_{f} }}$$

The thermophysical properties of a tri-hybrid nanofluid can be estimated using models from the literature.

The dimensionless variables listed below need to be used right away:

7 $$ \xi = \frac{x}{{\overset{\lower0.5em\hbox{$\smash{\scriptscriptstyle\frown}$}}{L} }},\, \, \,y = \frac{y}{{\overset{\lower0.5em\hbox{$\smash{\scriptscriptstyle\frown}$}}{L} }},\, \, \,u = \frac{{\tilde{U}}}{{V_{0} }}, \, \,v = \frac{{\tilde{V}}}{{V_{0} }}, \, \,\overset{\lower0.5em\hbox{$\smash{\scriptscriptstyle\frown}$}}{\theta } = \frac{{T - T_{c} }}{\Delta T},\,\,\,\,H = \frac{{\tilde{H}}}{{H_{0} }},\,\,\,\,t = \frac{{V_{0} }}{{\overset{\lower0.5em\hbox{$\smash{\scriptscriptstyle\frown}$}}{L} }}t^{\prime} $$

From Eqs. (4) and (5), we can deduce that;

8 $$ \begin{aligned} & \frac{\partial J}{{\partial t}} + u\frac{\partial J}{{\partial \xi }} + v\frac{\partial J}{{\partial \eta }} = \left( {1 - \overset{\lower0.5em\hbox{$\smash{\scriptscriptstyle\frown}$}}{\varphi }_{1} } \right)\left( {1 - \overset{\lower0.5em\hbox{$\smash{\scriptscriptstyle\frown}$}}{\varphi }_{2} + \overset{\lower0.5em\hbox{$\smash{\scriptscriptstyle\frown}$}}{\varphi }_{3} \left( {\overset{\lower0.5em\hbox{$\smash{\scriptscriptstyle\frown}$}}{\rho } s_{1} /\overset{\lower0.5em\hbox{$\smash{\scriptscriptstyle\frown}$}}{\rho }_{f} } \right) + \overset{\lower0.5em\hbox{$\smash{\scriptscriptstyle\frown}$}}{\varphi }_{2} \left( {\overset{\lower0.5em\hbox{$\smash{\scriptscriptstyle\frown}$}}{\rho } s_{2} /\overset{\lower0.5em\hbox{$\smash{\scriptscriptstyle\frown}$}}{\rho }_{f} } \right)} \right)\left( {1 - \overset{\lower0.5em\hbox{$\smash{\scriptscriptstyle\frown}$}}{\varphi }_{2} } \right)^{2.5} \left( {1 - \overset{\lower0.5em\hbox{$\smash{\scriptscriptstyle\frown}$}}{\varphi }_{3} } \right)^{2.5} \frac{1}{{{\text{Re}}}}\nabla^{2} J \\ & \quad + \frac{Mn}{{\left( {1 - \overset{\lower0.5em\hbox{$\smash{\scriptscriptstyle\frown}$}}{\varphi }_{2} } \right)\left( {1 - \overset{\lower0.5em\hbox{$\smash{\scriptscriptstyle\frown}$}}{\varphi }_{1} + \overset{\lower0.5em\hbox{$\smash{\scriptscriptstyle\frown}$}}{\varphi }_{2} \left( {\overset{\lower0.5em\hbox{$\smash{\scriptscriptstyle\frown}$}}{\rho } s_{1} /\overset{\lower0.5em\hbox{$\smash{\scriptscriptstyle\frown}$}}{\rho }_{f} } \right) + \overset{\lower0.5em\hbox{$\smash{\scriptscriptstyle\frown}$}}{\varphi }_{3} \left( {\overset{\lower0.5em\hbox{$\smash{\scriptscriptstyle\frown}$}}{\rho } s_{2} /\overset{\lower0.5em\hbox{$\smash{\scriptscriptstyle\frown}$}}{\rho }_{f} } \right)} \right)}}H\,\left( {\frac{\partial H}{{\partial \eta }}.\frac{{\partial \overset{\lower0.5em\hbox{$\smash{\scriptscriptstyle\frown}$}}{\theta } }}{\partial \xi } - \frac{\partial H}{{\partial \xi }}.\frac{{\partial \overset{\lower0.5em\hbox{$\smash{\scriptscriptstyle\frown}$}}{\theta } }}{\partial \eta }} \right), \\ \end{aligned} $$

9a $$  \begin{aligned}   \nabla ^{2} \overset{\lower0.5em\hbox{$\smash{\scriptscriptstyle\frown}$}}{\theta }  &  = \Pr *\left( {\frac{{\left( {1 - \overset{\lower0.5em\hbox{$\smash{\scriptscriptstyle\frown}$}}{\varphi } _{2} } \right)\left( {1 - \overset{\lower0.5em\hbox{$\smash{\scriptscriptstyle\frown}$}}{\varphi } _{1}  + \overset{\lower0.5em\hbox{$\smash{\scriptscriptstyle\frown}$}}{\varphi } _{2} \left( {\overset{\lower0.5em\hbox{$\smash{\scriptscriptstyle\frown}$}}{\rho } s_{1} /\overset{\lower0.5em\hbox{$\smash{\scriptscriptstyle\frown}$}}{\rho } _{f} } \right) + \overset{\lower0.5em\hbox{$\smash{\scriptscriptstyle\frown}$}}{\varphi } _{3} \left( {\overset{\lower0.5em\hbox{$\smash{\scriptscriptstyle\frown}$}}{\rho } s_{2} /\overset{\lower0.5em\hbox{$\smash{\scriptscriptstyle\frown}$}}{\rho } _{f} } \right)} \right)}}{{\left( {1 - \overset{\lower0.5em\hbox{$\smash{\scriptscriptstyle\frown}$}}{\varphi } _{1} } \right)^{{ - 2.5}} \left( {1 - \overset{\lower0.5em\hbox{$\smash{\scriptscriptstyle\frown}$}}{\varphi } _{2} } \right)^{{ - 2.5}} }}} \right) \\     & *\left( {1 - \overset{\lower0.5em\hbox{$\smash{\scriptscriptstyle\frown}$}}{\varphi } _{2} } \right)\left( {1 - \overset{\lower0.5em\hbox{$\smash{\scriptscriptstyle\frown}$}}{\varphi } _{1}  + \overset{\lower0.5em\hbox{$\smash{\scriptscriptstyle\frown}$}}{\varphi } _{2} \left( {\overset{\lower0.5em\hbox{$\smash{\scriptscriptstyle\frown}$}}{\rho } s_{1} *c_{p} \overset{\lower0.5em\hbox{$\smash{\scriptscriptstyle\frown}$}}{\rho } s_{1} /\overset{\lower0.5em\hbox{$\smash{\scriptscriptstyle\frown}$}}{\rho } _{f} *c_{{p} _{f}} } \right) + \overset{\lower0.5em\hbox{$\smash{\scriptscriptstyle\frown}$}}{\varphi } _{3} \left( {\overset{\lower0.5em\hbox{$\smash{\scriptscriptstyle\frown}$}}{\rho } s_{3} *c_{p} \overset{\lower0.5em\hbox{$\smash{\scriptscriptstyle\frown}$}}{\rho } s_{3} /\overset{\lower0.5em\hbox{$\smash{\scriptscriptstyle\frown}$}}{\rho } _{f} *c_{{p} _{f}} } \right)} \right) \\     & \text{Re} *\left( {\frac{{\left( {1 - \overset{\lower0.5em\hbox{$\smash{\scriptscriptstyle\frown}$}}{\varphi } _{1} } \right)^{{ - 2.5}} \left( {1 - \overset{\lower0.5em\hbox{$\smash{\scriptscriptstyle\frown}$}}{\varphi } _{2} } \right)^{{ - 2.5}} }}{{\left( {1 - \overset{\lower0.5em\hbox{$\smash{\scriptscriptstyle\frown}$}}{\varphi } _{2} } \right)\left( {1 - \overset{\lower0.5em\hbox{$\smash{\scriptscriptstyle\frown}$}}{\varphi } _{1}  + \overset{\lower0.5em\hbox{$\smash{\scriptscriptstyle\frown}$}}{\varphi } _{2} \left( {\overset{\lower0.5em\hbox{$\smash{\scriptscriptstyle\frown}$}}{\rho } s_{1} /\overset{\lower0.5em\hbox{$\smash{\scriptscriptstyle\frown}$}}{\rho } _{f} } \right) + \overset{\lower0.5em\hbox{$\smash{\scriptscriptstyle\frown}$}}{\varphi } _{2} \left( {\overset{\lower0.5em\hbox{$\smash{\scriptscriptstyle\frown}$}}{\rho } s_{3} /\overset{\lower0.5em\hbox{$\smash{\scriptscriptstyle\frown}$}}{\rho } _{f} } \right)} \right)}}} \right)\left\{ {\frac{{\partial \overset{\lower0.5em\hbox{$\smash{\scriptscriptstyle\frown}$}}{\theta } }}{{\partial \xi }}\frac{{\partial \tilde{\psi }}}{{\partial \eta }} - \frac{{\partial \overset{\lower0.5em\hbox{$\smash{\scriptscriptstyle\frown}$}}{\theta } }}{{\partial \eta }}\frac{{\partial \tilde{\psi }}}{{\partial \xi }}} \right\} \\     &  + \Pr *\left( {\frac{{\left( {1 - \overset{\lower0.5em\hbox{$\smash{\scriptscriptstyle\frown}$}}{\varphi } _{2} } \right)\left( {1 - \overset{\lower0.5em\hbox{$\smash{\scriptscriptstyle\frown}$}}{\varphi } _{1}  + \overset{\lower0.5em\hbox{$\smash{\scriptscriptstyle\frown}$}}{\varphi } _{2} \left( {\overset{\lower0.5em\hbox{$\smash{\scriptscriptstyle\frown}$}}{\rho } s_{1} /\overset{\lower0.5em\hbox{$\smash{\scriptscriptstyle\frown}$}}{\rho } _{f} } \right) + \overset{\lower0.5em\hbox{$\smash{\scriptscriptstyle\frown}$}}{\varphi } _{3} \left( {\overset{\lower0.5em\hbox{$\smash{\scriptscriptstyle\frown}$}}{\rho } s_{2} /\overset{\lower0.5em\hbox{$\smash{\scriptscriptstyle\frown}$}}{\rho } _{f} } \right)} \right)}}{{\left( {1 - \overset{\lower0.5em\hbox{$\smash{\scriptscriptstyle\frown}$}}{\varphi } _{1} } \right)^{{ - 2.5}} \left( {1 - \overset{\lower0.5em\hbox{$\smash{\scriptscriptstyle\frown}$}}{\varphi } _{2} } \right)^{{ - 2.5}} }}} \right) \\     & *\left( {1 - \overset{\lower0.5em\hbox{$\smash{\scriptscriptstyle\frown}$}}{\varphi } _{2} } \right)\left( {1 - \overset{\lower0.5em\hbox{$\smash{\scriptscriptstyle\frown}$}}{\varphi } _{1}  + \overset{\lower0.5em\hbox{$\smash{\scriptscriptstyle\frown}$}}{\varphi } _{1} \left( {\overset{\lower0.5em\hbox{$\smash{\scriptscriptstyle\frown}$}}{\rho } s_{1} *c_{p} \overset{\lower0.5em\hbox{$\smash{\scriptscriptstyle\frown}$}}{\rho } s_{1} /\overset{\lower0.5em\hbox{$\smash{\scriptscriptstyle\frown}$}}{\rho } _{f} *c_{{p} _{f}} } \right) + \overset{\lower0.5em\hbox{$\smash{\scriptscriptstyle\frown}$}}{\varphi } _{2} \left( {\overset{\lower0.5em\hbox{$\smash{\scriptscriptstyle\frown}$}}{\rho } s_{2} *c_{p} \overset{\lower0.5em\hbox{$\smash{\scriptscriptstyle\frown}$}}{\rho } s_{2} /\overset{\lower0.5em\hbox{$\smash{\scriptscriptstyle\frown}$}}{\rho } _{f} *c_{{p} _{f}} } \right)} \right) \\     & *\frac{{Mn}}{{\left( {1 - \overset{\lower0.5em\hbox{$\smash{\scriptscriptstyle\frown}$}}{\varphi } _{2} } \right)\left( {1 - \overset{\lower0.5em\hbox{$\smash{\scriptscriptstyle\frown}$}}{\varphi } _{1}  + \overset{\lower0.5em\hbox{$\smash{\scriptscriptstyle\frown}$}}{\varphi } _{1} \left( {\overset{\lower0.5em\hbox{$\smash{\scriptscriptstyle\frown}$}}{\rho } s_{1} /\overset{\lower0.5em\hbox{$\smash{\scriptscriptstyle\frown}$}}{\rho } _{f} } \right) + \overset{\lower0.5em\hbox{$\smash{\scriptscriptstyle\frown}$}}{\varphi } _{2} \left( {\overset{\lower0.5em\hbox{$\smash{\scriptscriptstyle\frown}$}}{\rho } s_{2} /\overset{\lower0.5em\hbox{$\smash{\scriptscriptstyle\frown}$}}{\rho } _{f} } \right)} \right)}} \\     & \text{Re} *\left( {\frac{{\left( {1 - \overset{\lower0.5em\hbox{$\smash{\scriptscriptstyle\frown}$}}{\varphi } _{1} } \right)^{{ - 2.5}} \left( {1 - \overset{\lower0.5em\hbox{$\smash{\scriptscriptstyle\frown}$}}{\varphi } _{3} } \right)^{{ - 2.5}} }}{{\left( {1 - \overset{\lower0.5em\hbox{$\smash{\scriptscriptstyle\frown}$}}{\varphi } _{2} } \right)\left( {1 - \overset{\lower0.5em\hbox{$\smash{\scriptscriptstyle\frown}$}}{\varphi } _{1}  + \overset{\lower0.5em\hbox{$\smash{\scriptscriptstyle\frown}$}}{\varphi } _{1} \left( {\overset{\lower0.5em\hbox{$\smash{\scriptscriptstyle\frown}$}}{\rho } s_{1} /\overset{\lower0.5em\hbox{$\smash{\scriptscriptstyle\frown}$}}{\rho } _{f} } \right) + \overset{\lower0.5em\hbox{$\smash{\scriptscriptstyle\frown}$}}{\varphi } _{3} \left( {\overset{\lower0.5em\hbox{$\smash{\scriptscriptstyle\frown}$}}{\rho } s_{3} /\overset{\lower0.5em\hbox{$\smash{\scriptscriptstyle\frown}$}}{\rho } _{f} } \right)} \right)}}} \right)EcH(\varepsilon  - \tilde{\psi })\left\{ {\frac{{\partial H}}{{\partial \xi }}\frac{{\partial \tilde{\psi }}}{{\partial \eta }} - \frac{{\partial H}}{{\partial \eta }}\frac{{\partial \tilde{\psi }}}{{\partial \xi }}} \right\} \\     &  + \Pr *\left( {\frac{{\left( {1 - \overset{\lower0.5em\hbox{$\smash{\scriptscriptstyle\frown}$}}{\varphi } _{2} } \right)\left( {1 - \overset{\lower0.5em\hbox{$\smash{\scriptscriptstyle\frown}$}}{\varphi } _{1}  + \overset{\lower0.5em\hbox{$\smash{\scriptscriptstyle\frown}$}}{\varphi } _{1} \left( {\overset{\lower0.5em\hbox{$\smash{\scriptscriptstyle\frown}$}}{\rho } s_{1} /\overset{\lower0.5em\hbox{$\smash{\scriptscriptstyle\frown}$}}{\rho } _{f} } \right) + \overset{\lower0.5em\hbox{$\smash{\scriptscriptstyle\frown}$}}{\varphi } _{3} \left( {\overset{\lower0.5em\hbox{$\smash{\scriptscriptstyle\frown}$}}{\rho } s_{2} /\overset{\lower0.5em\hbox{$\smash{\scriptscriptstyle\frown}$}}{\rho } _{f} } \right)} \right)}}{{\left( {1 - \overset{\lower0.5em\hbox{$\smash{\scriptscriptstyle\frown}$}}{\varphi } _{1} } \right)^{{ - 2.5}} \left( {1 - \overset{\lower0.5em\hbox{$\smash{\scriptscriptstyle\frown}$}}{\varphi } _{2} } \right)^{{ - 2.5}} }}} \right) \\     & *\left( {1 - \overset{\lower0.5em\hbox{$\smash{\scriptscriptstyle\frown}$}}{\varphi } _{2} } \right)\left( {1 - \overset{\lower0.5em\hbox{$\smash{\scriptscriptstyle\frown}$}}{\varphi } _{1}  + \overset{\lower0.5em\hbox{$\smash{\scriptscriptstyle\frown}$}}{\varphi } _{1} \left( {\overset{\lower0.5em\hbox{$\smash{\scriptscriptstyle\frown}$}}{\rho } s_{1} *c_{p} \overset{\lower0.5em\hbox{$\smash{\scriptscriptstyle\frown}$}}{\rho } s_{1} /\overset{\lower0.5em\hbox{$\smash{\scriptscriptstyle\frown}$}}{\rho } _{f} *c_{{p} _{f}} } \right) + \overset{\lower0.5em\hbox{$\smash{\scriptscriptstyle\frown}$}}{\varphi } _{2} \left( {\overset{\lower0.5em\hbox{$\smash{\scriptscriptstyle\frown}$}}{\rho } s_{2} *c_{p} \overset{\lower0.5em\hbox{$\smash{\scriptscriptstyle\frown}$}}{\rho } s_{2} /\overset{\lower0.5em\hbox{$\smash{\scriptscriptstyle\frown}$}}{\rho } _{f} *c_{{p} _{f}} } \right)} \right) \\     & Ec\left\{ {\left( {\frac{{\partial ^{2} \tilde{\psi }}}{{\partial \eta ^{2} }} - \frac{{\partial ^{2} \tilde{\psi }}}{{\partial \xi ^{2} }}} \right)^{2}  + 4\left( {\frac{{\partial ^{2} \tilde{\psi }}}{{\partial \xi \partial \eta }}} \right)^{2} } \right\}, \\  \end{aligned}   $$

where

9b $$ H_{1} (\xi ,\eta ) = H_{0} \left\{ {\tanh A^{\prime}_{1} \left( {\xi - \xi_{1} } \right) - \tanh A^{\prime}_{2} \left( {\xi - \xi_{2} } \right)} \right\}, $$

$$ \begin{aligned} H_{2} (\xi ,\eta ) & = H_{0} \left\{ {\tanh A_{1} \left( {\xi - \xi_{3} } \right) - \tanh A_{2} \left( {\xi - \xi_{4} } \right)} \right\} \\ H_{3} (\xi ,\eta ) & = H_{0} \left\{ {\tanh A_{1} \left( {\xi - \xi_{5} } \right) - \tanh A_{2} \left( {\xi - \xi_{6} } \right)} \right\}, \\ H_{4} (\xi ,\eta ) & = H_{0} \left\{ {\tanh A^{\prime}_{1} \left( {\eta - \eta_{1} } \right) - \tanh A^{\prime}_{2} \left( {\eta - \eta_{2} } \right)} \right\}, \\ H_{5} (\xi ,\eta ) & = H_{0} \left\{ {\tanh A_{1} \left( {\eta - \eta_{3} } \right) - \tanh A_{2} \left( {\eta - \eta_{4} } \right)} \right\} \\ H_{6} (\xi ,\eta ) & = H_{0} \left\{ {\tanh A_{1} \left( {\eta - \eta_{5} } \right) - \tanh A_{2} \left( {\eta - \eta_{6} } \right)} \right\} \\ \end{aligned} $$

in the strips defined by $\,\xi_{1} \le \xi \le \xi_{2} ,\,\,\xi_{3} \le \xi \le \xi_{4} ,\,\,\xi_{5} \le \xi \le \xi_{6} ;\,\,\,\,0 \le \eta \le 1$ and $\eta_{1} \le \eta \le \eta_{2} ,\,\,\,\,\eta_{3} \le \eta \le \eta_{4} ,\,\,\,\,\eta_{5} \le \eta \le \eta_{6} ;\,\,\,\,0 \le \xi \le 1$, respectively.

Finally,

9c $$ H\left( {\xi ,\eta } \right) = H_{1} \left( {\xi ,\eta } \right) + H_{2} \left( {\xi ,\eta } \right) + H_{3} \left( {\xi ,\eta } \right) + H_{4} \left( {\xi ,\eta } \right) + H_{5} \left( {\xi ,\eta } \right) + H_{6} \left( {\xi ,\eta } \right) $$

The stream-vorticity formulation is used to modify Eqs. (1)–(4) with the magnetic field and electric potential terms. The equations below show this formulation.

10 $$ \overset{\lower0.5em\hbox{$\smash{\scriptscriptstyle\frown}$}}{u} = \frac{{\partial \tilde{\psi }}}{\partial \eta },\, \, \overset{\lower0.5em\hbox{$\smash{\scriptscriptstyle\frown}$}}{v} = \frac{{\partial \tilde{\psi }}}{\partial \xi }\quad {\text{and}}\quad \,\left( {\frac{{\partial \overset{\lower0.5em\hbox{$\smash{\scriptscriptstyle\frown}$}}{u} }}{\partial \eta } - \frac{{\partial \overset{\lower0.5em\hbox{$\smash{\scriptscriptstyle\frown}$}}{v} }}{\partial \xi }} \right) = - \overset{\lower0.5em\hbox{$\smash{\scriptscriptstyle\frown}$}}{\omega } \quad {\text{or}}\quad \left\{ {\left( {\frac{{\partial^{2} \tilde{\psi }}}{{\partial \xi^{2} }} + \frac{{\partial^{2} \tilde{\psi }}}{{\partial \eta^{2} }}} \right) = - \overset{\lower0.5em\hbox{$\smash{\scriptscriptstyle\frown}$}}{\omega } } \right\}. $$

The dimensionless boundary conditions are given by:

*Left and Right Vertical Walls (Adiabatic):*

11a $$ \,\overset{\lower0.5em\hbox{$\smash{\scriptscriptstyle\frown}$}}{u} (0,\,\eta ) = \overset{\lower0.5em\hbox{$\smash{\scriptscriptstyle\frown}$}}{u} (1,\,\eta ) = 0,\,\,\,\,\,\,\,\,\left( {\frac{\partial \theta }{{\partial \xi }}} \right)_{\xi = 0} = 0, \, \left( {\frac{\partial \theta }{{\partial \xi }}} \right)_{\xi = 1} = 0,\,\,\,\,\,\,\,\,\overset{\lower0.5em\hbox{$\smash{\scriptscriptstyle\frown}$}}{v} (0,\,\eta ) = \overset{\lower0.5em\hbox{$\smash{\scriptscriptstyle\frown}$}}{v} (1,\,\eta ) = 0,\,\,\,\,\,\,\,\,\,\,0 < \eta < 1 $$

*Upper Horizontal Wall:*

11b $$ \overset{\lower0.5em\hbox{$\smash{\scriptscriptstyle\frown}$}}{u} (\xi ,\,1) = 0,\,\,\,\,\,\,\, \, \overset{\lower0.5em\hbox{$\smash{\scriptscriptstyle\frown}$}}{\theta } (\xi ,\,1) = 0,\,\,\,\,\,\,\,\,\,\overset{\lower0.5em\hbox{$\smash{\scriptscriptstyle\frown}$}}{v} (\xi ,\,1) = 0;\,\,\,\,\,\,\,\,\,\,\,\,\,\,\,0 < \xi < 1 $$

*Lower Horizontal Wall:*

11c $$ \overset{\lower0.5em\hbox{$\smash{\scriptscriptstyle\frown}$}}{u} (\xi ,\,0) = - 1,\,\,\,\,\,\,\,\,\overset{\lower0.5em\hbox{$\smash{\scriptscriptstyle\frown}$}}{\theta } (\xi ,\,0) = 1,\,\,\,\,\,\,\,\overset{\lower0.5em\hbox{$\smash{\scriptscriptstyle\frown}$}}{\text{v}}(\xi ,\,0) = 0;\,\,\,\,\,\,\,\,\,\,\,\,0 < \xi < 1. $$

### Physical quantities of interest

Fluid dynamics, the study of how fluids (liquids and gases) behave and interact under various conditions, involves the analysis of a wide range of physical quantities to understand the behavior of fluids. In this work, we are interested in studying the heat transfer (*Nu*) and the wall shear stress (*CfRe*).

*Nusselt Number*: The Nusselt number (*Nu*) is a dimensionless quantity used in the field of heat transfer. It relates the convective heat transfer rate in a fluid to the conductive heat transfer rate through a stationary medium. In other words, it helps us understand how effectively heat is being transferred from a solid surface to a fluid that flows over or around it. The Nusselt number takes into account the effect of convection, which enhances heat transfer due to the fluid motion.

Mathematically, the Nusselt number is defined as:

$$ {\text{Nu}} = \frac{{\overset{\lower0.5em\hbox{$\smash{\scriptscriptstyle\frown}$}}{q} L}}{{k_{hnf} \Delta T}} $$

where,

$$ \overset{\lower0.5em\hbox{$\smash{\scriptscriptstyle\frown}$}}{q} = \left. { - k_{hnf} \left( { \frac{\partial T}{{\partial \eta }}} \right)} \right|_{\eta = 0} \quad {\text{ is a heat flux}}{.} $$

- $\Delta T$ is the temperature difference between both horizontal plates.
- $L$ is a characteristic length related to the geometry of the system.
- $k_{hnf}$ is the thermal conductivity of tri-hybrid nanofluid.

Using the dimensionless variables, we get:

$$ \left. {N_{u} = \frac{{k_{thnf} }}{{k_{nf} }}\frac{{\partial \overset{\lower0.5em\hbox{$\smash{\scriptscriptstyle\frown}$}}{\theta } }}{\partial \eta }} \right|_{\eta = 0} $$

A higher Nusselt number indicates more efficient heat transfer. It's often used to correlate and predict heat transfer coefficients in various situations, such as fluid flow over a solid surface, heat exchangers, and other convective heat transfer scenarios.

*Skin Friction:* Skin friction is a term used in fluid dynamics to describe the frictional force per unit area acting on a fluid as it flows over a solid surface. It's a crucial concept in understanding the resistance a fluid encounters when moving across a surface. Skin friction is primarily responsible for the drag force experienced by objects moving through a fluid, like an aircraft through air or a ship through water.

Mathematically, skin friction is related to the shear stress $\left( \tau \right)$ at the solid–fluid interface:

$$ \left. {{\text{CfRe}} = \frac{2\tau }{{\overset{\lower0.5em\hbox{$\smash{\scriptscriptstyle\frown}$}}{\rho }_{hnf} v_{0}^{2} }}} \right|_{\eta = 0} $$

where

$$ \left. {\tau = \tilde{\mu }_{hnf} \left( {\frac{{ \partial \overset{\lower0.5em\hbox{$\smash{\scriptscriptstyle\frown}$}}{U} }}{\partial \eta }} \right)} \right|_{\eta = 0} $$

Using the dimensionless variables, we get:

$$ CfRe = 2\frac{{\left[ {\,\,\,\left( {1\, - \,\overset{\lower0.5em\hbox{$\smash{\scriptscriptstyle\frown}$}}{\varphi }_{1} } \right)\,\,\left( {1\, - \,\overset{\lower0.5em\hbox{$\smash{\scriptscriptstyle\frown}$}}{\varphi } 2} \right)\,\,\left( {1\, - \,\overset{\lower0.5em\hbox{$\smash{\scriptscriptstyle\frown}$}}{\varphi }_{3} } \right)\,\,\,} \right]^{2.5} }}{{\left[ {\left( {1 - \overset{\lower0.5em\hbox{$\smash{\scriptscriptstyle\frown}$}}{\varphi }_{3} } \right)\left\{ {\left( {1 - \overset{\lower0.5em\hbox{$\smash{\scriptscriptstyle\frown}$}}{\varphi }_{2} } \right)\left( {1 - \overset{\lower0.5em\hbox{$\smash{\scriptscriptstyle\frown}$}}{\varphi }_{1} + \overset{\lower0.5em\hbox{$\smash{\scriptscriptstyle\frown}$}}{\varphi }_{1} \frac{{\overset{\lower0.5em\hbox{$\smash{\scriptscriptstyle\frown}$}}{\rho }_{s1} }}{{\overset{\lower0.5em\hbox{$\smash{\scriptscriptstyle\frown}$}}{\rho }_{f} }}} \right) + \overset{\lower0.5em\hbox{$\smash{\scriptscriptstyle\frown}$}}{\varphi }_{2} \frac{{\overset{\lower0.5em\hbox{$\smash{\scriptscriptstyle\frown}$}}{\rho }_{s2} }}{{\overset{\lower0.5em\hbox{$\smash{\scriptscriptstyle\frown}$}}{\rho }_{f} }}} \right\} + \overset{\lower0.5em\hbox{$\smash{\scriptscriptstyle\frown}$}}{\varphi }_{3} \frac{{\overset{\lower0.5em\hbox{$\smash{\scriptscriptstyle\frown}$}}{\rho }_{s3} }}{{\overset{\lower0.5em\hbox{$\smash{\scriptscriptstyle\frown}$}}{\rho }_{f} }}} \right]}}\frac{{\partial \overset{\lower0.5em\hbox{$\smash{\scriptscriptstyle\frown}$}}{u} }}{\partial y} $$

Higher skin friction values indicate greater resistance to fluid flow and higher drag forces. Reducing skin friction is an important consideration in the design of streamlined surfaces to improve the efficiency of vehicles and reduce energy consumption.

In short, the Nusselt number quantifies convective heat transfer efficiency, while skin friction measures the drag force and resistance encountered by a fluid as it flows over a solid surface. The Nusselt number and skin friction are indispensable tools for engineers and researchers in a wide range of fields from heat exchangers to aerodynamics. They provide insights into heat transfer efficiency, fluid dynamics, and the design of energy-efficient systems. By leveraging these concepts, engineers can optimize designs, improve energy efficiency, and enhance the performance of various engineering applications.

## Alternating direction implicit (ADI) technique

A numerical method to solve the Eqs. (4), (5), and (6) that describe the fluid flow and heat transfer in a cavity has been employed. It solves the equations in one direction at a time, while keeping the other direction fixed. This makes the solution faster and more stable. The method also uses central difference, which means using the average values of the nearby points to estimate the change at a point. This is a good and accurate way to change the equations into algebraic equations on a grid. The method we use is a type of finite difference method, which is a general way to solve governing equations. Finite differences can also help us find changes from data that are not continuous. One of the benefits of the finite difference method is that it is easy to do and can be very accurate by using more points in the stencil, which is a group of points that we use to calculate the change at a point.

The steps for solving the equations are:

1. Solve the fluid flow equation in the horizontal direction, while keeping the vertical direction fixed.
2. Solve the heat transfer equation in the horizontal direction, while keeping the vertical direction fixed.
3. Solve the fluid flow equation in the vertical direction, using the solution from step 1.
4. Solve the heat transfer equation in the vertical direction, using the solution from step 2.

We repeat these steps until we get the final solution.

12 $$  \begin{aligned}   \frac{{\overset{\lower0.5em\hbox{$\smash{\scriptscriptstyle\frown}$}}{w} _{{i,j}}^{{\left( {n + 1/2} \right)}}  - \overset{\lower0.5em\hbox{$\smash{\scriptscriptstyle\frown}$}}{w} _{{i,j}}^{{\left( n \right)}} }}{{{\raise0.7ex\hbox{${\delta t}$} \!\mathord{\left/ {\vphantom {{\delta t} 2}}\right.\kern-\nulldelimiterspace} \!\lower0.7ex\hbox{$2$}}}} &  = \text{Re} *\left( {\frac{{\left( {1 - \overset{\lower0.5em\hbox{$\smash{\scriptscriptstyle\frown}$}}{\varphi } _{1} } \right)^{{ - 2.5}} \left( {1 - \overset{\lower0.5em\hbox{$\smash{\scriptscriptstyle\frown}$}}{\varphi } _{2} } \right)^{{ - 2.5}} \left( {1 - \overset{\lower0.5em\hbox{$\smash{\scriptscriptstyle\frown}$}}{\varphi } _{3} } \right)^{{ - 2.5}} }}{{\left( {\left( {1 - \overset{\lower0.5em\hbox{$\smash{\scriptscriptstyle\frown}$}}{\varphi } _{3} } \right)\left[ {\left( {1 - \overset{\lower0.5em\hbox{$\smash{\scriptscriptstyle\frown}$}}{\varphi } _{1} } \right)\left\{ {\left( {1 - \overset{\lower0.5em\hbox{$\smash{\scriptscriptstyle\frown}$}}{\varphi } _{2} } \right)\overset{\lower0.5em\hbox{$\smash{\scriptscriptstyle\frown}$}}{\rho } _{f}  + \overset{\lower0.5em\hbox{$\smash{\scriptscriptstyle\frown}$}}{\varphi } _{1} \overset{\lower0.5em\hbox{$\smash{\scriptscriptstyle\frown}$}}{\rho } _{{s1}} /\overset{\lower0.5em\hbox{$\smash{\scriptscriptstyle\frown}$}}{\rho } _{f} } \right\} + \overset{\lower0.5em\hbox{$\smash{\scriptscriptstyle\frown}$}}{\varphi } _{2} \overset{\lower0.5em\hbox{$\smash{\scriptscriptstyle\frown}$}}{\rho } _{{s2}} /\overset{\lower0.5em\hbox{$\smash{\scriptscriptstyle\frown}$}}{\rho } _{f} } \right] + \overset{\lower0.5em\hbox{$\smash{\scriptscriptstyle\frown}$}}{\varphi } _{3} \overset{\lower0.5em\hbox{$\smash{\scriptscriptstyle\frown}$}}{\rho } _{{s3}} /\overset{\lower0.5em\hbox{$\smash{\scriptscriptstyle\frown}$}}{\rho } _{f} } \right)}}} \right) \\     & \quad \left\{ {\frac{{\overset{\lower0.5em\hbox{$\smash{\scriptscriptstyle\frown}$}}{w} _{{i - 1,j}}^{{\left( {n + 1/2} \right)}}  - 2\overset{\lower0.5em\hbox{$\smash{\scriptscriptstyle\frown}$}}{w} _{{i,j}}^{{\left( {n + 1/2} \right)}}  + \overset{\lower0.5em\hbox{$\smash{\scriptscriptstyle\frown}$}}{w} _{{i + 1,j}}^{{\left( {n + 1/2} \right)}} }}{{h^{2} }} + \frac{{\overset{\lower0.5em\hbox{$\smash{\scriptscriptstyle\frown}$}}{w} _{{i,j - 1}}^{{\left( n \right)}}  - 2\overset{\lower0.5em\hbox{$\smash{\scriptscriptstyle\frown}$}}{w} _{{i,j}}^{{\left( n \right)}}  + \overset{\lower0.5em\hbox{$\smash{\scriptscriptstyle\frown}$}}{w} _{{i,j + 1}}^{{\left( n \right)}} }}{{k^{2} }}} \right\} \\     & \quad  + \frac{{Mn}}{{\left( {\left( {1 - \overset{\lower0.5em\hbox{$\smash{\scriptscriptstyle\frown}$}}{\varphi } _{3} } \right)\left[ {\left( {1 - \overset{\lower0.5em\hbox{$\smash{\scriptscriptstyle\frown}$}}{\varphi } _{1} } \right)\left\{ {\left( {1 - \overset{\lower0.5em\hbox{$\smash{\scriptscriptstyle\frown}$}}{\varphi } _{2} } \right)\overset{\lower0.5em\hbox{$\smash{\scriptscriptstyle\frown}$}}{\rho } _{f}  + \overset{\lower0.5em\hbox{$\smash{\scriptscriptstyle\frown}$}}{\varphi } _{1} \overset{\lower0.5em\hbox{$\smash{\scriptscriptstyle\frown}$}}{\rho } _{{s1}} /\overset{\lower0.5em\hbox{$\smash{\scriptscriptstyle\frown}$}}{\rho } _{f} } \right\} + \overset{\lower0.5em\hbox{$\smash{\scriptscriptstyle\frown}$}}{\varphi } _{2} \overset{\lower0.5em\hbox{$\smash{\scriptscriptstyle\frown}$}}{\rho } _{{s2}} /\overset{\lower0.5em\hbox{$\smash{\scriptscriptstyle\frown}$}}{\rho } _{f} } \right] + \overset{\lower0.5em\hbox{$\smash{\scriptscriptstyle\frown}$}}{\varphi } _{3} \overset{\lower0.5em\hbox{$\smash{\scriptscriptstyle\frown}$}}{\rho } _{{s3}} /\overset{\lower0.5em\hbox{$\smash{\scriptscriptstyle\frown}$}}{\rho } _{f} } \right)}} \\     & \quad H_{{i,j}} \left\{ {\frac{{H_{{i,j + 1}}  - H_{{i,j - 1}} }}{{2k}}\frac{{\overset{\lower0.5em\hbox{$\smash{\scriptscriptstyle\frown}$}}{\theta } _{{i + 1,j}}^{{\left( n \right)}}  - \overset{\lower0.5em\hbox{$\smash{\scriptscriptstyle\frown}$}}{\theta } _{{i - 1,j}}^{{\left( n \right)}} }}{{2h}} - \frac{{H_{{i + 1,j}}  - H_{{i - 1,j}} }}{{2h}}\frac{{\overset{\lower0.5em\hbox{$\smash{\scriptscriptstyle\frown}$}}{\theta } _{{i,j + 1}}^{{\left( n \right)}}  - \overset{\lower0.5em\hbox{$\smash{\scriptscriptstyle\frown}$}}{\theta } _{{i,j - 1}}^{{\left( n \right)}} }}{{2k}}} \right\} \\     & \quad  - \overset{\lower0.5em\hbox{$\smash{\scriptscriptstyle\frown}$}}{u} _{{i,j}}^{{\left( {n + {1 \mathord{\left/ {\vphantom {1 2}} \right. \kern-\nulldelimiterspace} 2}} \right)}} \left( {\frac{{\overset{\lower0.5em\hbox{$\smash{\scriptscriptstyle\frown}$}}{w} _{{i + 1,j}}^{{\left( {n + 1} \right)}}  - \overset{\lower0.5em\hbox{$\smash{\scriptscriptstyle\frown}$}}{w} _{{i - 1,j}}^{{\left( {n + 1} \right)}} }}{{2h}}} \right) - \overset{\lower0.5em\hbox{$\smash{\scriptscriptstyle\frown}$}}{v} _{{i,j}}^{{\left( {n + {1 \mathord{\left/ {\vphantom {1 2}} \right. \kern-\nulldelimiterspace} 2}} \right)}} \left( {\frac{{\overset{\lower0.5em\hbox{$\smash{\scriptscriptstyle\frown}$}}{w} _{{i,j + 1}}^{{\left( n \right)}}  - \overset{\lower0.5em\hbox{$\smash{\scriptscriptstyle\frown}$}}{w} _{{i,j - 1}}^{{\left( n \right)}} }}{{2k}}} \right) \\  \end{aligned}       $$

13 $$ \begin{aligned}   \frac{{\overset{\lower0.5em\hbox{$\smash{\scriptscriptstyle\frown}$}}{\theta } _{{i,j}}^{{\left( {n + {1 \mathord{\left/ {\vphantom {1 2}} \right. \kern-\nulldelimiterspace} 2}} \right)}}  - \overset{\lower0.5em\hbox{$\smash{\scriptscriptstyle\frown}$}}{\theta } _{{i,j}}^{{\left( n \right)}} }}{{\delta t/2}} &  = \left\{ {\frac{{\overset{\lower0.5em\hbox{$\smash{\scriptscriptstyle\frown}$}}{\theta } _{{i - 1,j}}^{{\left( {n + 1/2} \right)}}  - 2\overset{\lower0.5em\hbox{$\smash{\scriptscriptstyle\frown}$}}{\theta } _{{i,j}}^{{\left( {n + 1/2} \right)}}  + \overset{\lower0.5em\hbox{$\smash{\scriptscriptstyle\frown}$}}{\theta } _{{i + 1,j}}^{{\left( {n + {1 \mathord{\left/ {\vphantom {1 2}} \right. \kern-\nulldelimiterspace} 2}} \right)}} }}{{h^{2} }} + \frac{{\overset{\lower0.5em\hbox{$\smash{\scriptscriptstyle\frown}$}}{\theta } _{{i,j - 1}}^{{\left( n \right)}}  + \overset{\lower0.5em\hbox{$\smash{\scriptscriptstyle\frown}$}}{\theta } _{{i,j + 1}}^{{\left( n \right)}}  - 2\overset{\lower0.5em\hbox{$\smash{\scriptscriptstyle\frown}$}}{\theta } _{{i,j}}^{{\left( n \right)}} }}{{k^{2} }}} \right\} \\     & \quad  + \Pr *\left( {\frac{{\left( {\left( {1 - \overset{\lower0.5em\hbox{$\smash{\scriptscriptstyle\frown}$}}{\varphi } _{3} } \right)\left[ {\left( {1 - \overset{\lower0.5em\hbox{$\smash{\scriptscriptstyle\frown}$}}{\varphi } _{1} } \right)\left\{ {\left( {1 - \overset{\lower0.5em\hbox{$\smash{\scriptscriptstyle\frown}$}}{\varphi } _{2} } \right)\overset{\lower0.5em\hbox{$\smash{\scriptscriptstyle\frown}$}}{\rho } _{f}  + \overset{\lower0.5em\hbox{$\smash{\scriptscriptstyle\frown}$}}{\varphi } _{1} \overset{\lower0.5em\hbox{$\smash{\scriptscriptstyle\frown}$}}{\rho } _{{s1}} /\overset{\lower0.5em\hbox{$\smash{\scriptscriptstyle\frown}$}}{\rho } _{f} } \right\} + \overset{\lower0.5em\hbox{$\smash{\scriptscriptstyle\frown}$}}{\varphi } _{2} \overset{\lower0.5em\hbox{$\smash{\scriptscriptstyle\frown}$}}{\rho } _{{s2}} /\overset{\lower0.5em\hbox{$\smash{\scriptscriptstyle\frown}$}}{\rho } _{f} } \right] + \overset{\lower0.5em\hbox{$\smash{\scriptscriptstyle\frown}$}}{\varphi } _{3} \overset{\lower0.5em\hbox{$\smash{\scriptscriptstyle\frown}$}}{\rho } _{{s3}} /\overset{\lower0.5em\hbox{$\smash{\scriptscriptstyle\frown}$}}{\rho } _{f} } \right)}}{{\left( {1 - \overset{\lower0.5em\hbox{$\smash{\scriptscriptstyle\frown}$}}{\varphi } _{1} } \right)^{{ - 2.5}} \left( {1 - \overset{\lower0.5em\hbox{$\smash{\scriptscriptstyle\frown}$}}{\varphi } _{2} } \right)^{{ - 2.5}} \left( {1 - \overset{\lower0.5em\hbox{$\smash{\scriptscriptstyle\frown}$}}{\varphi } _{3} } \right)^{{ - 2.5}} }}} \right) \\     & \quad *\left( {\left( {1 - \overset{\lower0.5em\hbox{$\smash{\scriptscriptstyle\frown}$}}{\varphi } _{3} } \right)\left[ {\left( {1 - \overset{\lower0.5em\hbox{$\smash{\scriptscriptstyle\frown}$}}{\varphi } _{1} } \right)\left\{ {\left( {1 - \overset{\lower0.5em\hbox{$\smash{\scriptscriptstyle\frown}$}}{\varphi } _{2} } \right)\overset{\lower0.5em\hbox{$\smash{\scriptscriptstyle\frown}$}}{\rho } _{f}  + \overset{\lower0.5em\hbox{$\smash{\scriptscriptstyle\frown}$}}{\varphi } _{1} c_{{p_{{S1}} }} \overset{\lower0.5em\hbox{$\smash{\scriptscriptstyle\frown}$}}{\rho } _{{s1}} /\overset{\lower0.5em\hbox{$\smash{\scriptscriptstyle\frown}$}}{\rho } _{f} } \right\} + \overset{\lower0.5em\hbox{$\smash{\scriptscriptstyle\frown}$}}{\varphi } _{2} c_{{p_{{S2}} }} \overset{\lower0.5em\hbox{$\smash{\scriptscriptstyle\frown}$}}{\rho } _{{s2}} /\overset{\lower0.5em\hbox{$\smash{\scriptscriptstyle\frown}$}}{\rho } _{f} } \right] + \overset{\lower0.5em\hbox{$\smash{\scriptscriptstyle\frown}$}}{\varphi } _{3} c_{{p_{{S3}} }} \overset{\lower0.5em\hbox{$\smash{\scriptscriptstyle\frown}$}}{\rho } _{{s3}} /\overset{\lower0.5em\hbox{$\smash{\scriptscriptstyle\frown}$}}{\rho } _{f} } \right) \\     & \quad *\frac{{Mn}}{{\left( {\left( {1 - \overset{\lower0.5em\hbox{$\smash{\scriptscriptstyle\frown}$}}{\varphi } _{3} } \right)\left[ {\left( {1 - \overset{\lower0.5em\hbox{$\smash{\scriptscriptstyle\frown}$}}{\varphi } _{1} } \right)\left\{ {\left( {1 - \overset{\lower0.5em\hbox{$\smash{\scriptscriptstyle\frown}$}}{\varphi } _{2} } \right)\overset{\lower0.5em\hbox{$\smash{\scriptscriptstyle\frown}$}}{\rho } _{f}  + \overset{\lower0.5em\hbox{$\smash{\scriptscriptstyle\frown}$}}{\varphi } _{1} \overset{\lower0.5em\hbox{$\smash{\scriptscriptstyle\frown}$}}{\rho } _{{s1}} /\overset{\lower0.5em\hbox{$\smash{\scriptscriptstyle\frown}$}}{\rho } _{f} } \right\} + \overset{\lower0.5em\hbox{$\smash{\scriptscriptstyle\frown}$}}{\varphi } _{2} \overset{\lower0.5em\hbox{$\smash{\scriptscriptstyle\frown}$}}{\rho } _{{s2}} /\overset{\lower0.5em\hbox{$\smash{\scriptscriptstyle\frown}$}}{\rho } _{f} } \right] + \overset{\lower0.5em\hbox{$\smash{\scriptscriptstyle\frown}$}}{\varphi } _{3} \overset{\lower0.5em\hbox{$\smash{\scriptscriptstyle\frown}$}}{\rho } _{{s3}} /\overset{\lower0.5em\hbox{$\smash{\scriptscriptstyle\frown}$}}{\rho } _{f} } \right)}} \\     & \quad \text{Re} *\left( {\frac{{\left( {1 - \overset{\lower0.5em\hbox{$\smash{\scriptscriptstyle\frown}$}}{\varphi } _{1} } \right)^{{ - 2.5}} \left( {1 - \overset{\lower0.5em\hbox{$\smash{\scriptscriptstyle\frown}$}}{\varphi } _{2} } \right)^{{ - 2.5}} \left( {1 - \overset{\lower0.5em\hbox{$\smash{\scriptscriptstyle\frown}$}}{\varphi } _{3} } \right)^{{ - 2.5}} }}{{\left( {1 - \overset{\lower0.5em\hbox{$\smash{\scriptscriptstyle\frown}$}}{\varphi } _{2} } \right)\left( {1 - \overset{\lower0.5em\hbox{$\smash{\scriptscriptstyle\frown}$}}{\varphi } _{1}  + \overset{\lower0.5em\hbox{$\smash{\scriptscriptstyle\frown}$}}{\varphi } _{1} \left( {\overset{\lower0.5em\hbox{$\smash{\scriptscriptstyle\frown}$}}{\rho } s_{1} /\overset{\lower0.5em\hbox{$\smash{\scriptscriptstyle\frown}$}}{\rho } _{f} } \right) + \overset{\lower0.5em\hbox{$\smash{\scriptscriptstyle\frown}$}}{\varphi } _{2} \left( {\overset{\lower0.5em\hbox{$\smash{\scriptscriptstyle\frown}$}}{\rho } s_{2} /\overset{\lower0.5em\hbox{$\smash{\scriptscriptstyle\frown}$}}{\rho } _{f} } \right)} \right)}}} \right) \\     & \quad H_{{i,j}} \left( {\tilde{\psi }_{{i,j}}  - \varepsilon } \right)\left\{ {\overset{\lower0.5em\hbox{$\smash{\scriptscriptstyle\frown}$}}{u} _{{i,j}}^{{\left( {n + 1/2} \right)}} \frac{{H_{{i,j + 1}}  - H_{{i,j - 1}} }}{{2k}} + \overset{\lower0.5em\hbox{$\smash{\scriptscriptstyle\frown}$}}{v} _{{i,j}}^{{\left( {n + 1/2} \right)}} \frac{{H_{{i + 1,j}}  - H_{{i - 1,j}} }}{{2h}}} \right\} \\     & \quad  - \overset{\lower0.5em\hbox{$\smash{\scriptscriptstyle\frown}$}}{u} _{{i,j}}^{{\left( {n + {1 \mathord{\left/ {\vphantom {1 2}} \right. \kern-\nulldelimiterspace} 2}} \right)}} \left( {\frac{{\overset{\lower0.5em\hbox{$\smash{\scriptscriptstyle\frown}$}}{\theta } _{{i + 1,j}}^{{\left( {n + {1 \mathord{\left/ {\vphantom {1 2}} \right. \kern-\nulldelimiterspace} 2}} \right)}}  - \overset{\lower0.5em\hbox{$\smash{\scriptscriptstyle\frown}$}}{\theta } _{{i - 1,j}}^{{\left( {n + {1 \mathord{\left/ {\vphantom {1 2}} \right. \kern-\nulldelimiterspace} 2}} \right)}} }}{{2h}}} \right) - \overset{\lower0.5em\hbox{$\smash{\scriptscriptstyle\frown}$}}{v} _{{i,j}}^{{\left( {n + {1 \mathord{\left/ {\vphantom {1 2}} \right. \kern-\nulldelimiterspace} 2}} \right)}} \left( {\frac{{\overset{\lower0.5em\hbox{$\smash{\scriptscriptstyle\frown}$}}{\theta } _{{i,j + 1}}^{{\left( n \right)}}  - \overset{\lower0.5em\hbox{$\smash{\scriptscriptstyle\frown}$}}{\theta } _{{i,j - 1}}^{{\left( n \right)}} }}{{2k}}} \right) -  \\     & \quad \Pr *\left( {\frac{{\left( {\left( {1 - \overset{\lower0.5em\hbox{$\smash{\scriptscriptstyle\frown}$}}{\varphi } _{3} } \right)\left[ {\left( {1 - \overset{\lower0.5em\hbox{$\smash{\scriptscriptstyle\frown}$}}{\varphi } _{1} } \right)\left\{ {\left( {1 - \overset{\lower0.5em\hbox{$\smash{\scriptscriptstyle\frown}$}}{\varphi } _{2} } \right)\overset{\lower0.5em\hbox{$\smash{\scriptscriptstyle\frown}$}}{\rho } _{f}  + \overset{\lower0.5em\hbox{$\smash{\scriptscriptstyle\frown}$}}{\varphi } _{1} \overset{\lower0.5em\hbox{$\smash{\scriptscriptstyle\frown}$}}{\rho } _{{s1}} /\overset{\lower0.5em\hbox{$\smash{\scriptscriptstyle\frown}$}}{\rho } _{f} } \right\} + \overset{\lower0.5em\hbox{$\smash{\scriptscriptstyle\frown}$}}{\varphi } _{2} \overset{\lower0.5em\hbox{$\smash{\scriptscriptstyle\frown}$}}{\rho } _{{s2}} /\overset{\lower0.5em\hbox{$\smash{\scriptscriptstyle\frown}$}}{\rho } _{f} } \right] + \overset{\lower0.5em\hbox{$\smash{\scriptscriptstyle\frown}$}}{\varphi } _{3} \overset{\lower0.5em\hbox{$\smash{\scriptscriptstyle\frown}$}}{\rho } _{{s3}} /\overset{\lower0.5em\hbox{$\smash{\scriptscriptstyle\frown}$}}{\rho } _{f} } \right)}}{{\left( {1 - \overset{\lower0.5em\hbox{$\smash{\scriptscriptstyle\frown}$}}{\varphi } _{1} } \right)^{{ - 2.5}} \left( {1 - \overset{\lower0.5em\hbox{$\smash{\scriptscriptstyle\frown}$}}{\varphi } _{2} } \right)^{{ - 2.5}} }}} \right) \\     & \quad *\left( {\left( {1 - \overset{\lower0.5em\hbox{$\smash{\scriptscriptstyle\frown}$}}{\varphi } _{3} } \right)\left[ {\left( {1 - \overset{\lower0.5em\hbox{$\smash{\scriptscriptstyle\frown}$}}{\varphi } _{1} } \right)\left\{ {\left( {1 - \overset{\lower0.5em\hbox{$\smash{\scriptscriptstyle\frown}$}}{\varphi } _{2} } \right)\overset{\lower0.5em\hbox{$\smash{\scriptscriptstyle\frown}$}}{\rho } _{f}  + \overset{\lower0.5em\hbox{$\smash{\scriptscriptstyle\frown}$}}{\varphi } _{1} c_{{p_{{S1}} }} \overset{\lower0.5em\hbox{$\smash{\scriptscriptstyle\frown}$}}{\rho } _{{s1}} /\overset{\lower0.5em\hbox{$\smash{\scriptscriptstyle\frown}$}}{\rho } _{f} } \right\} + \overset{\lower0.5em\hbox{$\smash{\scriptscriptstyle\frown}$}}{\varphi } _{2} c_{{p_{{S2}} }} \overset{\lower0.5em\hbox{$\smash{\scriptscriptstyle\frown}$}}{\rho } _{{s2}} /\overset{\lower0.5em\hbox{$\smash{\scriptscriptstyle\frown}$}}{\rho } _{f} } \right] + \overset{\lower0.5em\hbox{$\smash{\scriptscriptstyle\frown}$}}{\varphi } _{3} c_{{p_{{S3}} }} \overset{\lower0.5em\hbox{$\smash{\scriptscriptstyle\frown}$}}{\rho } _{{s3}} /\overset{\lower0.5em\hbox{$\smash{\scriptscriptstyle\frown}$}}{\rho } _{f} } \right) \\     & \quad 
Ec\left\{ {\left( {\frac{{\overset{\lower0.5em\hbox{$\smash{\scriptscriptstyle\frown}$}}{u} _{{i,j + 1}}^{{\left( {n + 1/2} \right)}}  - \overset{\lower0.5em\hbox{$\smash{\scriptscriptstyle\frown}$}}{u} _{{i,j - 1}}^{{\left( {n + 1/2} \right)}} }}{{2k}} + \frac{{\overset{\lower0.5em\hbox{$\smash{\scriptscriptstyle\frown}$}}{v} _{{i + 1,j}}^{{\left( {n + 1/2} \right)}}  - \overset{\lower0.5em\hbox{$\smash{\scriptscriptstyle\frown}$}}{v} _{{i - 1,j}}^{{\left( {n + 1/2} \right)}} }}{{2h}}} \right)^{2}  + 4\left( {\frac{{\overset{\lower0.5em\hbox{$\smash{\scriptscriptstyle\frown}$}}{u} _{{i + 1,j}}^{{\left( {n + 1/2} \right)}}  - \overset{\lower0.5em\hbox{$\smash{\scriptscriptstyle\frown}$}}{u} _{{i - 1,j}}^{{\left( {n + 1/2} \right)}} }}{{2h}}} \right)^{2} } \right\} \\  \end{aligned}  $$

14 $$ \frac{{\tilde{\psi }_{i - 1,j}^{{\left( {n + 1} \right)}} + \tilde{\psi }_{i + 1,j}^{{\left( {n + 1} \right)}} - 2\tilde{\psi }_{i,j}^{{\left( {n + 1} \right)}} }}{{h^{2} }} + \frac{{\tilde{\psi }_{i,j - 1}^{{\left( {n + 1} \right)}} + \tilde{\psi }_{i,j + 1}^{{\left( {n + 1} \right)}} - 2\tilde{\psi }_{i,j}^{{\left( {n + 1} \right)}} }}{{k^{2} }} = - \overset{\lower0.5em\hbox{$\smash{\scriptscriptstyle\frown}$}}{w}_{i,j}^{{\left( {n + {1 \mathord{\left/ {\vphantom {1 2}} \right. \kern-0pt} 2}} \right)}} , $$

15 $$ \overset{\lower0.5em\hbox{$\smash{\scriptscriptstyle\frown}$}}{u}_{i,j}^{{\left( {n + 1} \right)}} = \frac{{ - \tilde{\psi }_{i,j - 1}^{{\left( {n + 1} \right)}} + \tilde{\psi }_{i,j + 1}^{{\left( {n + 1} \right)}} }}{2k}, $$

16 $$ \overset{\lower0.5em\hbox{$\smash{\scriptscriptstyle\frown}$}}{v}_{i,j}^{{\left( {n + 1} \right)}} = - \frac{{\tilde{\psi }_{i + 1,j}^{{\left( {n + 1} \right)}} - \tilde{\psi }_{i - 1,j}^{{\left( {n + 1} \right)}} }}{2h}, $$

17 $$ \begin{aligned}   \frac{{\overset{\lower0.5em\hbox{$\smash{\scriptscriptstyle\frown}$}}{w} _{{i,j}}^{{\left( {n + 1} \right)}}  - \overset{\lower0.5em\hbox{$\smash{\scriptscriptstyle\frown}$}}{w} _{{i,j}}^{{\left( {n + 1/2} \right)}} }}{{{\raise0.7ex\hbox{${\delta t}$} \!\mathord{\left/ {\vphantom {{\delta t} 2}}\right.\kern-\nulldelimiterspace} \!\lower0.7ex\hbox{$2$}}}} &  = \text{Re} *\left( {\frac{{\left( {1 - \overset{\lower0.5em\hbox{$\smash{\scriptscriptstyle\frown}$}}{\varphi } _{1} } \right)^{{ - 2.5}} \left( {1 - \overset{\lower0.5em\hbox{$\smash{\scriptscriptstyle\frown}$}}{\varphi } _{2} } \right)^{{ - 2.5}} \left( {1 - \overset{\lower0.5em\hbox{$\smash{\scriptscriptstyle\frown}$}}{\varphi } _{3} } \right)^{{ - 2.5}} }}{{\left( {\left( {1 - \overset{\lower0.5em\hbox{$\smash{\scriptscriptstyle\frown}$}}{\varphi } _{3} } \right)\left[ {\left( {1 - \overset{\lower0.5em\hbox{$\smash{\scriptscriptstyle\frown}$}}{\varphi } _{1} } \right)\left\{ {\left( {1 - \overset{\lower0.5em\hbox{$\smash{\scriptscriptstyle\frown}$}}{\varphi } _{2} } \right)\overset{\lower0.5em\hbox{$\smash{\scriptscriptstyle\frown}$}}{\rho } _{f}  + \overset{\lower0.5em\hbox{$\smash{\scriptscriptstyle\frown}$}}{\varphi } _{1} \overset{\lower0.5em\hbox{$\smash{\scriptscriptstyle\frown}$}}{\rho } _{{s1}} /\overset{\lower0.5em\hbox{$\smash{\scriptscriptstyle\frown}$}}{\rho } _{f} } \right\} + \overset{\lower0.5em\hbox{$\smash{\scriptscriptstyle\frown}$}}{\varphi } _{2} \overset{\lower0.5em\hbox{$\smash{\scriptscriptstyle\frown}$}}{\rho } _{{s2}} /\overset{\lower0.5em\hbox{$\smash{\scriptscriptstyle\frown}$}}{\rho } _{f} } \right] + \overset{\lower0.5em\hbox{$\smash{\scriptscriptstyle\frown}$}}{\varphi } _{3} \overset{\lower0.5em\hbox{$\smash{\scriptscriptstyle\frown}$}}{\rho } _{{s3}} /\overset{\lower0.5em\hbox{$\smash{\scriptscriptstyle\frown}$}}{\rho } _{f} } \right)}}} \right) \\     & \quad \left\{ {\frac{{\overset{\lower0.5em\hbox{$\smash{\scriptscriptstyle\frown}$}}{w} _{{i - 1,j}}^{{\left( {n + {1 \mathord{\left/ {\vphantom {1 2}} \right. \kern-\nulldelimiterspace} 2}} \right)}}  - 2\overset{\lower0.5em\hbox{$\smash{\scriptscriptstyle\frown}$}}{w} _{{i,j}}^{{\left( {n + {1 \mathord{\left/ {\vphantom {1 2}} \right. \kern-\nulldelimiterspace} 2}} \right)}}  + \overset{\lower0.5em\hbox{$\smash{\scriptscriptstyle\frown}$}}{w} _{{i + 1,j}}^{{\left( {n + {1 \mathord{\left/ {\vphantom {1 2}} \right. \kern-\nulldelimiterspace} 2}} \right)}} }}{{h^{2} }} + \frac{{\overset{\lower0.5em\hbox{$\smash{\scriptscriptstyle\frown}$}}{w} _{{i,j - 1}}^{{\left( {n + 1} \right)}}  - 2\overset{\lower0.5em\hbox{$\smash{\scriptscriptstyle\frown}$}}{w} _{{i,j}}^{{\left( {n + 1} \right)}}  + \overset{\lower0.5em\hbox{$\smash{\scriptscriptstyle\frown}$}}{w} _{{i,j + 1}}^{{\left( {n + 1} \right)}} }}{{k^{2} }}} \right\} \\     & \quad  + \frac{{Mn}}{{\left( {\left( {1 - \overset{\lower0.5em\hbox{$\smash{\scriptscriptstyle\frown}$}}{\varphi } _{3} } \right)\left[ {\left( {1 - \overset{\lower0.5em\hbox{$\smash{\scriptscriptstyle\frown}$}}{\varphi } _{1} } \right)\left\{ {\left( {1 - \overset{\lower0.5em\hbox{$\smash{\scriptscriptstyle\frown}$}}{\varphi } _{2} } \right)\overset{\lower0.5em\hbox{$\smash{\scriptscriptstyle\frown}$}}{\rho } _{f}  + \overset{\lower0.5em\hbox{$\smash{\scriptscriptstyle\frown}$}}{\varphi } _{1} \overset{\lower0.5em\hbox{$\smash{\scriptscriptstyle\frown}$}}{\rho } _{{s1}} /\overset{\lower0.5em\hbox{$\smash{\scriptscriptstyle\frown}$}}{\rho } _{f} } \right\} + \overset{\lower0.5em\hbox{$\smash{\scriptscriptstyle\frown}$}}{\varphi } _{2} \overset{\lower0.5em\hbox{$\smash{\scriptscriptstyle\frown}$}}{\rho } _{{s2}} /\overset{\lower0.5em\hbox{$\smash{\scriptscriptstyle\frown}$}}{\rho } _{f} } \right] + \overset{\lower0.5em\hbox{$\smash{\scriptscriptstyle\frown}$}}{\varphi } _{3} \overset{\lower0.5em\hbox{$\smash{\scriptscriptstyle\frown}$}}{\rho } _{{s3}} /\overset{\lower0.5em\hbox{$\smash{\scriptscriptstyle\frown}$}}{\rho } _{f} } \right)}} \\     & \quad H_{{i,j}} \left\{ {\frac{{H_{{i,j + 1}}  - H_{{i,j - 1}} }}{{2k}}\frac{{\overset{\lower0.5em\hbox{$\smash{\scriptscriptstyle\frown}$}}{\theta } _{{i + 1,j}}^{{\left( {n + 1} \right)}}  - \overset{\lower0.5em\hbox{$\smash{\scriptscriptstyle\frown}$}}{\theta } _{{i - 1,j}}^{{\left( {n + 1} \right)}} }}{{2h}} - \frac{{H_{{i + 1,j}}  - H_{{i - 1,j}} }}{{2h}}\frac{{\overset{\lower0.5em\hbox{$\smash{\scriptscriptstyle\frown}$}}{\theta } _{{i,j + 1}}^{{\left( {n + 1} \right)}}  - \overset{\lower0.5em\hbox{$\smash{\scriptscriptstyle\frown}$}}{\theta } _{{i,j - 1}}^{{\left( {n + 1} \right)}} }}{{2k}}} \right\} \\     & \quad  - \overset{\lower0.5em\hbox{$\smash{\scriptscriptstyle\frown}$}}{u} _{{i,j}}^{{\left( {n + {1 \mathord{\left/ {\vphantom {1 2}} \right. \kern-\nulldelimiterspace} 2}} \right)}} \left( {\frac{{\overset{\lower0.5em\hbox{$\smash{\scriptscriptstyle\frown}$}}{w} _{{i + 1,j}}^{{\left( {n + 1} \right)}}  - \overset{\lower0.5em\hbox{$\smash{\scriptscriptstyle\frown}$}}{w} _{{i - 1,j}}^{{\left( {n + 1} \right)}} }}{{2h}}} \right) - \overset{\lower0.5em\hbox{$\smash{\scriptscriptstyle\frown}$}}{v} _{{i,j}}^{{\left( {n + {1 \mathord{\left/ {\vphantom {1 2}} \right. \kern-\nulldelimiterspace} 2}} \right)}} \left( {\frac{{\overset{\lower0.5em\hbox{$\smash{\scriptscriptstyle\frown}$}}{w} _{{i,j + 1}}^{{\left( {n + 1} \right)}}  - \overset{\lower0.5em\hbox{$\smash{\scriptscriptstyle\frown}$}}{w} _{{i,j - 1}}^{{\left( {n + 1} \right)}} }}{{2k}}} \right) \\  \end{aligned}  $$

18 $$ \begin{aligned}    & \frac{{\overset{\lower0.5em\hbox{$\smash{\scriptscriptstyle\frown}$}}{\theta } _{{i,j}}^{{\left( {n + 1} \right)}}  - \overset{\lower0.5em\hbox{$\smash{\scriptscriptstyle\frown}$}}{\theta } _{{i,j}}^{{\left( {\left( {n + 1/2} \right)} \right)}} }}{{\delta t/2}} = \left\{ {\frac{{\overset{\lower0.5em\hbox{$\smash{\scriptscriptstyle\frown}$}}{\theta } _{{i - 1,j}}^{{\left( {n + 1/2} \right)}}  - 2\overset{\lower0.5em\hbox{$\smash{\scriptscriptstyle\frown}$}}{\theta } _{{i,j}}^{{\left( {n + 1/2} \right)}}  + \overset{\lower0.5em\hbox{$\smash{\scriptscriptstyle\frown}$}}{\theta } _{{i + 1,j}}^{{\left( {n + 1/2} \right)}} }}{{h^{2} }} + \frac{{\overset{\lower0.5em\hbox{$\smash{\scriptscriptstyle\frown}$}}{\theta } _{{i,j - 1}}^{{\left( {n + 1} \right)}}  + \overset{\lower0.5em\hbox{$\smash{\scriptscriptstyle\frown}$}}{\theta } _{{i,j + 1}}^{{\left( {n + 1} \right)}}  - 2\overset{\lower0.5em\hbox{$\smash{\scriptscriptstyle\frown}$}}{\theta } _{{i,j}}^{{\left( {n + 1} \right)}} }}{{k^{2} }}} \right\} \\     & \quad  + \Pr *\left( {\frac{{\left( {\left( {1 - \overset{\lower0.5em\hbox{$\smash{\scriptscriptstyle\frown}$}}{\varphi } _{3} } \right)\left[ {\left( {1 - \overset{\lower0.5em\hbox{$\smash{\scriptscriptstyle\frown}$}}{\varphi } _{1} } \right)\left\{ {\left( {1 - \overset{\lower0.5em\hbox{$\smash{\scriptscriptstyle\frown}$}}{\varphi } _{2} } \right)\overset{\lower0.5em\hbox{$\smash{\scriptscriptstyle\frown}$}}{\rho } _{f}  + \overset{\lower0.5em\hbox{$\smash{\scriptscriptstyle\frown}$}}{\varphi } _{1} \overset{\lower0.5em\hbox{$\smash{\scriptscriptstyle\frown}$}}{\rho } _{{s1}} /\overset{\lower0.5em\hbox{$\smash{\scriptscriptstyle\frown}$}}{\rho } _{f} } \right\} + \overset{\lower0.5em\hbox{$\smash{\scriptscriptstyle\frown}$}}{\varphi } _{2} \overset{\lower0.5em\hbox{$\smash{\scriptscriptstyle\frown}$}}{\rho } _{{s2}} /\overset{\lower0.5em\hbox{$\smash{\scriptscriptstyle\frown}$}}{\rho } _{f} } \right] + \overset{\lower0.5em\hbox{$\smash{\scriptscriptstyle\frown}$}}{\varphi } _{3} \overset{\lower0.5em\hbox{$\smash{\scriptscriptstyle\frown}$}}{\rho } _{{s3}} /\overset{\lower0.5em\hbox{$\smash{\scriptscriptstyle\frown}$}}{\rho } _{f} } \right)}}{{\left( {1 - \overset{\lower0.5em\hbox{$\smash{\scriptscriptstyle\frown}$}}{\varphi } _{1} } \right)^{{ - 2.5}} \left( {1 - \overset{\lower0.5em\hbox{$\smash{\scriptscriptstyle\frown}$}}{\varphi } _{2} } \right)^{{ - 2.5}} \left( {1 - \overset{\lower0.5em\hbox{$\smash{\scriptscriptstyle\frown}$}}{\varphi } _{3} } \right)^{{ - 2.5}} }}} \right) \\     & \quad *\left( {\left( {1 - \overset{\lower0.5em\hbox{$\smash{\scriptscriptstyle\frown}$}}{\varphi } _{3} } \right)\left[ {\left( {1 - \overset{\lower0.5em\hbox{$\smash{\scriptscriptstyle\frown}$}}{\varphi } _{1} } \right)\left\{ {\left( {1 - \overset{\lower0.5em\hbox{$\smash{\scriptscriptstyle\frown}$}}{\varphi } _{2} } \right)\overset{\lower0.5em\hbox{$\smash{\scriptscriptstyle\frown}$}}{\rho } _{f}  + \overset{\lower0.5em\hbox{$\smash{\scriptscriptstyle\frown}$}}{\varphi } _{1} c_{{p_{{S1}} }} \overset{\lower0.5em\hbox{$\smash{\scriptscriptstyle\frown}$}}{\rho } _{{s1}} /\overset{\lower0.5em\hbox{$\smash{\scriptscriptstyle\frown}$}}{\rho } _{f} } \right\} + \overset{\lower0.5em\hbox{$\smash{\scriptscriptstyle\frown}$}}{\varphi } _{2} c_{{p_{{S2}} }} \overset{\lower0.5em\hbox{$\smash{\scriptscriptstyle\frown}$}}{\rho } _{{s2}} /\overset{\lower0.5em\hbox{$\smash{\scriptscriptstyle\frown}$}}{\rho } _{f} } \right] + \overset{\lower0.5em\hbox{$\smash{\scriptscriptstyle\frown}$}}{\varphi } _{3} c_{{p_{{S3}} }} \overset{\lower0.5em\hbox{$\smash{\scriptscriptstyle\frown}$}}{\rho } _{{s3}} /\overset{\lower0.5em\hbox{$\smash{\scriptscriptstyle\frown}$}}{\rho } _{f} } \right) \\     & \quad *\frac{{Mn}}{{\left( {\left( {1 - \overset{\lower0.5em\hbox{$\smash{\scriptscriptstyle\frown}$}}{\varphi } _{3} } \right)\left[ {\left( {1 - \overset{\lower0.5em\hbox{$\smash{\scriptscriptstyle\frown}$}}{\varphi } _{1} } \right)\left\{ {\left( {1 - \overset{\lower0.5em\hbox{$\smash{\scriptscriptstyle\frown}$}}{\varphi } _{2} } \right)\overset{\lower0.5em\hbox{$\smash{\scriptscriptstyle\frown}$}}{\rho } _{f}  + \overset{\lower0.5em\hbox{$\smash{\scriptscriptstyle\frown}$}}{\varphi } _{1} \overset{\lower0.5em\hbox{$\smash{\scriptscriptstyle\frown}$}}{\rho } _{{s1}} /\overset{\lower0.5em\hbox{$\smash{\scriptscriptstyle\frown}$}}{\rho } _{f} } \right\} + \overset{\lower0.5em\hbox{$\smash{\scriptscriptstyle\frown}$}}{\varphi } _{2} \overset{\lower0.5em\hbox{$\smash{\scriptscriptstyle\frown}$}}{\rho } _{{s2}} /\overset{\lower0.5em\hbox{$\smash{\scriptscriptstyle\frown}$}}{\rho } _{f} } \right] + \overset{\lower0.5em\hbox{$\smash{\scriptscriptstyle\frown}$}}{\varphi } _{3} \overset{\lower0.5em\hbox{$\smash{\scriptscriptstyle\frown}$}}{\rho } _{{s3}} /\overset{\lower0.5em\hbox{$\smash{\scriptscriptstyle\frown}$}}{\rho } _{f} } \right)}} \\     & \quad \text{Re} *\left( {\frac{{\left( {1 - \overset{\lower0.5em\hbox{$\smash{\scriptscriptstyle\frown}$}}{\varphi } _{1} } \right)^{{ - 2.5}} \left( {1 - \overset{\lower0.5em\hbox{$\smash{\scriptscriptstyle\frown}$}}{\varphi } _{2} } \right)^{{ - 2.5}} \left( {1 - \overset{\lower0.5em\hbox{$\smash{\scriptscriptstyle\frown}$}}{\varphi } _{3} } \right)^{{ - 2.5}} }}{{\left( {\left( {1 - \overset{\lower0.5em\hbox{$\smash{\scriptscriptstyle\frown}$}}{\varphi } _{3} } \right)\left[ {\left( {1 - \overset{\lower0.5em\hbox{$\smash{\scriptscriptstyle\frown}$}}{\varphi } _{1} } \right)\left\{ {\left( {1 - \overset{\lower0.5em\hbox{$\smash{\scriptscriptstyle\frown}$}}{\varphi } _{2} } \right)\overset{\lower0.5em\hbox{$\smash{\scriptscriptstyle\frown}$}}{\rho } _{f}  + \overset{\lower0.5em\hbox{$\smash{\scriptscriptstyle\frown}$}}{\varphi } _{1} \overset{\lower0.5em\hbox{$\smash{\scriptscriptstyle\frown}$}}{\rho } _{{s1}} /\overset{\lower0.5em\hbox{$\smash{\scriptscriptstyle\frown}$}}{\rho } _{f} } \right\} + \overset{\lower0.5em\hbox{$\smash{\scriptscriptstyle\frown}$}}{\varphi } _{2} \overset{\lower0.5em\hbox{$\smash{\scriptscriptstyle\frown}$}}{\rho } _{{s2}} /\overset{\lower0.5em\hbox{$\smash{\scriptscriptstyle\frown}$}}{\rho } _{f} } \right] + \overset{\lower0.5em\hbox{$\smash{\scriptscriptstyle\frown}$}}{\varphi } _{3} \overset{\lower0.5em\hbox{$\smash{\scriptscriptstyle\frown}$}}{\rho } _{{s3}} /\overset{\lower0.5em\hbox{$\smash{\scriptscriptstyle\frown}$}}{\rho } _{f} } \right)}}} \right) \\     & \quad H_{{i,j}} \left( {\tilde{\psi }_{{i,j}}  - \varepsilon } \right)\left\{ {\overset{\lower0.5em\hbox{$\smash{\scriptscriptstyle\frown}$}}{u} _{{i,j}}^{{\left( {n + 1/2} \right)}} \frac{{H_{{i,j + 1}}  - H_{{i,j - 1}} }}{{2k}} + \overset{\lower0.5em\hbox{$\smash{\scriptscriptstyle\frown}$}}{v} _{{i,j}}^{{\left( {n + 1/2} \right)}} \frac{{H_{{i + 1,j}}  - H_{{i - 1,j}} }}{{2h}}} \right\} \\     & \quad  - \overset{\lower0.5em\hbox{$\smash{\scriptscriptstyle\frown}$}}{u} _{{i,j}}^{{\left( {n + {1 \mathord{\left/ {\vphantom {1 2}} \right. \kern-\nulldelimiterspace} 2}} \right)}} \left( {\frac{{\overset{\lower0.5em\hbox{$\smash{\scriptscriptstyle\frown}$}}{\theta } _{{i + 1,j}}^{{\left( {n + 1/2} \right)}}  - \overset{\lower0.5em\hbox{$\smash{\scriptscriptstyle\frown}$}}{\theta } _{{i - 1,j}}^{{\left( {n + 1/2} \right)}} }}{{2h}}} \right) - \overset{\lower0.5em\hbox{$\smash{\scriptscriptstyle\frown}$}}{v} _{{i,j}}^{{\left( {n + {1 \mathord{\left/ {\vphantom {1 2}} \right. \kern-\nulldelimiterspace} 2}} \right)}} \left( {\frac{{\overset{\lower0.5em\hbox{$\smash{\scriptscriptstyle\frown}$}}{\theta } _{{i,j + 1}}^{{\left( {n + 1} \right)}}  - \overset{\lower0.5em\hbox{$\smash{\scriptscriptstyle\frown}$}}{\theta } _{{i,j - 1}}^{{\left( {n + 1} \right)}} }}{{2k}}} \right) \\ & \quad - \Pr *\left( {\frac{{\left( {1 - \overset{\lower0.5em\hbox{$\smash{\scriptscriptstyle\frown}$}}{\varphi } _{2} } \right)\left( {1 - \overset{\lower0.5em\hbox{$\smash{\scriptscriptstyle\frown}$}}{\varphi } _{1}  + \overset{\lower0.5em\hbox{$\smash{\scriptscriptstyle\frown}$}}{\varphi } _{1} \left( {\overset{\lower0.5em\hbox{$\smash{\scriptscriptstyle\frown}$}}{\rho } s_{1} /\overset{\lower0.5em\hbox{$\smash{\scriptscriptstyle\frown}$}}{\rho } _{f} } \right) + \overset{\lower0.5em\hbox{$\smash{\scriptscriptstyle\frown}$}}{\varphi } _{2} \left( {\overset{\lower0.5em\hbox{$\smash{\scriptscriptstyle\frown}$}}{\rho } s_{2} /\overset{\lower0.5em\hbox{$\smash{\scriptscriptstyle\frown}$}}{\rho } _{f} } \right)} \right)}}{{\left( {1 - \overset{\lower0.5em\hbox{$\smash{\scriptscriptstyle\frown}$}}{\varphi } _{1} } \right)^{{ - 2.5}} \left( {1 - \overset{\lower0.5em\hbox{$\smash{\scriptscriptstyle\frown}$}}{\varphi } _{2} } \right)^{{ - 2.5}} }}} \right) \\     & \quad *\left( {\left( {1 - \overset{\lower0.5em\hbox{$\smash{\scriptscriptstyle\frown}$}}{\varphi } _{3} } \right)\left[ {\left( {1 - \overset{\lower0.5em\hbox{$\smash{\scriptscriptstyle\frown}$}}{\varphi } _{1} } \right)\left\{ {\left( {1 - \overset{\lower0.5em\hbox{$\smash{\scriptscriptstyle\frown}$}}{\varphi } _{2} } \right)\overset{\lower0.5em\hbox{$\smash{\scriptscriptstyle\frown}$}}{\rho } _{f}  + \overset{\lower0.5em\hbox{$\smash{\scriptscriptstyle\frown}$}}{\varphi } _{1} c_{{p_{{S1}} }} \overset{\lower0.5em\hbox{$\smash{\scriptscriptstyle\frown}$}}{\rho } _{{s1}} /\overset{\lower0.5em\hbox{$\smash{\scriptscriptstyle\frown}$}}{\rho } _{f} } \right\} + \overset{\lower0.5em\hbox{$\smash{\scriptscriptstyle\frown}$}}{\varphi } _{2} c_{{p_{{S2}} }} \overset{\lower0.5em\hbox{$\smash{\scriptscriptstyle\frown}$}}{\rho } _{{s2}} /\overset{\lower0.5em\hbox{$\smash{\scriptscriptstyle\frown}$}}{\rho } _{f} } \right] + \overset{\lower0.5em\hbox{$\smash{\scriptscriptstyle\frown}$}}{\varphi } _{3} c_{{p_{{S3}} }} \overset{\lower0.5em\hbox{$\smash{\scriptscriptstyle\frown}$}}{\rho } _{{s3}} /\overset{\lower0.5em\hbox{$\smash{\scriptscriptstyle\frown}$}}{\rho } _{f} } \right) \\     & \quad Ec\left\{ {\left( {\frac{{\overset{\lower0.5em\hbox{$\smash{\scriptscriptstyle\frown}$}}{u} _{{i,j + 1}}^{{\left( {n + 1/2} \right)}}  - \overset{\lower0.5em\hbox{$\smash{\scriptscriptstyle\frown}$}}{u} _{{i,j - 1}}^{{\left( {n + 1/2} 
\right)}} }}{{2k}} + \frac{{\overset{\lower0.5em\hbox{$\smash{\scriptscriptstyle\frown}$}}{v} _{{i + 1,j}}^{{\left( {n + 1/2} \right)}}  - \overset{\lower0.5em\hbox{$\smash{\scriptscriptstyle\frown}$}}{v} _{{i - 1,j}}^{{\left( {n + 1/2} \right)}} }}{{2h}}} \right)^{2}  + 4\left( {\frac{{\overset{\lower0.5em\hbox{$\smash{\scriptscriptstyle\frown}$}}{u} _{{i + 1,j}}^{{\left( {n + 1/2} \right)}}  - \overset{\lower0.5em\hbox{$\smash{\scriptscriptstyle\frown}$}}{u} _{{i - 1,j}}^{{\left( {n + 1/2} \right)}} }}{{2h}}} \right)^{2} } \right\} \\  \end{aligned}  $$

We stop the iteration scheme when we reach the criterion:

$$ \max \left\{ {abs\left( {\tilde{\psi }_{i,j}^{{\left( {n + 1} \right)}} - \tilde{\psi }_{i,j}^{\left( n \right)} } \right),\,abs\left( {w_{i,j}^{{\left( {n + 1} \right)}} - w_{i,j}^{\left( n \right)} } \right),\,abs\left( {\theta_{i,j}^{{\left( {n + 1} \right)}} - \theta_{i,j}^{\left( n \right)} } \right)} \right\} < TOL $$

This means that we have found a steady-state solution. We set *TOL* < 10^–6^ for this study.

### Flow chart of our numerical approach

**Figure 2:**
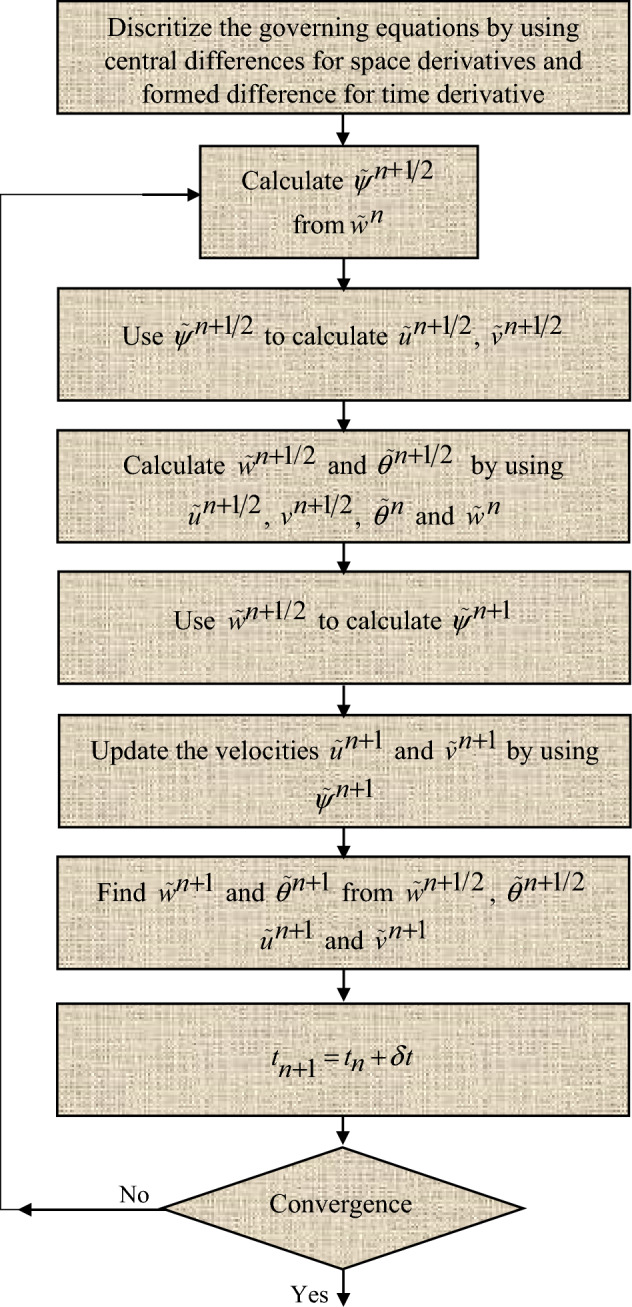
A flow Chart of Pseudo-Transient Approach.

Figure 2 shows the computational model for the pseudo-transient technique.

### Benchmarking the numerical scheme

**Figure 3:**
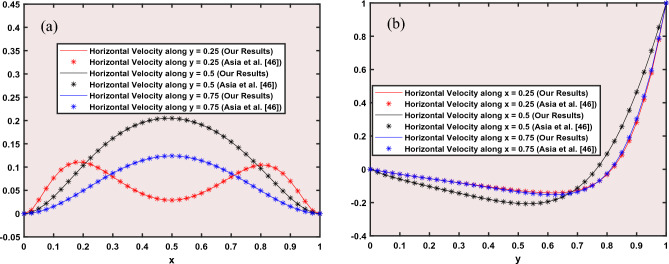
Numerical Findings Compared to Shih and Tan's Analytical Solution^46^.

To verify the reliability and accuracy of our numerical approach, we conduct a rigorous validation process. Our benchmark for comparison is the reputable work of Asia et al.^45^, whose findings provide a solid reference. We focus our attention on the horizontal velocity profiles, which clearly show the oscillations in the horizontal component of fluid velocity along a vertical axis. In our investigation, we examine a remarkable limiting scenario where several influential factors are set to zero: i.e. ($$\overset{\lower0.5em\hbox{$\smash{\scriptscriptstyle\frown}$}}{\varphi }_{1}$$ = $$\overset{\lower0.5em\hbox{$\smash{\scriptscriptstyle\frown}$}}{\varphi }_{2}$$ = 0, $${\text{Mn}}$$ = 0). In this fascinating domain, the fluid’s motion is driven only by the motion of one of the cavity’s lids. Our analysis takes place along three different horizontal lines, strategically located at y = 0.25, 0.50, and 0.75. Here, ‘y’ denotes the vertical coordinates, carefully normalized by the height of the lid-driven cavity, as shown in the informative Fig. 3a. The horizontal velocity along the cross-section defined by x = 0.25, 0.50, and 0.75 is given in Fig. 3b, by using the present approach as well as Asia et al.^45^. It the obvious to note that exact comparison validates our technique.

To validate our code, we compared it to the well-known problem of natural convection within a cavity. This problem has been previously studied by Chen et al.^47^ and Davis^48^, who used the Lattice Boltzmann method and finite difference method, respectively. Table 3 shows that the Nusselt number values obtained from our numerical analysis are in good agreement with those reported in these prior studies.

**Table 3:** Our results versus literature: a quality analysis.

Re	Average Nu for the Hot Wall		
	Lattice Boltzmann's approach^47^	Finite Difference approach (D. Davis^48^)	Our Approach
$10^{3}$	1.1192	1.1181	1.1182
$10^{4}$	2.2531	2.2432	2.2481

In this investigation, we use water as the basic base fluid, enriched with a mixture of nanoparticles including Ag, Al_2_O_3_, and TiO_2_. These nanoparticles are cleverly integrated into the base fluid to increase thermal conductivity and enable efficient heat transfer. In our simulations, we choose the value ε = 0.02 to represent the thermophysical characteristics of this remarkable nanofluid. The properties of water underlying our simulations are based on Pr. = 6.2.

To provide a context for our work, we introduce two important dimensionless parameters:

- The Reynolds number is a key indicator measuring the balance between inertial and viscous forces in fluid flow.
- The Eckert number is another vital dimensionless parameter that describes the relationship between kinetic energy and enthalpy change in fluid flow.

When the Reynolds number is very small, it results in a very low Eckert number (e.g., 10^–5^), indicating the dominance of viscous forces while kinetic energy becomes negligible. For a comprehensive understanding, Table 4 presents a detailed overview of the fundamental properties of both nanomaterials and the base fluid used in our research. These properties include density, particle size, specific heat, thermal conductivity, and viscosity, highlighting the foundation upon which our exploration rests.

**Table 4:** Thermal and physical features of water and nanomaterials (Ag–Al_2_O_3_–TiO_2_).

	$$C_{p} \left( {Jkg^{ - 1} K^{ - 1} } \right)$$	$$\sigma \left( {S \times m^{ - 1} } \right)$$	$$\overset{\lower0.5em\hbox{$\smash{\scriptscriptstyle\frown}$}}{\rho } \left( {kgm^{ - 3} } \right)$$	$$\beta \left( {K^{ - 1} } \right)$$	$$k\left( {Wm^{ - 1} K^{ - 1} } \right)$$
Water	4179	0.05	997.1	$21 \times 10^{ - 5}$	0.613
Silver(Ag)	235	$3.6 \times 10^{7}$	8933	$1.89 \times 10^{ - 5}$	429
Aluminum Oxide (Al_2_O_3_)	765	$1 \times 10^{ - 1}$	3970	$0.85 \times 10^{ - 5}$	40
Titanium Dioxide (TiO_2_)	686	$1 \times 10^{ - 12}$	5200	$0.90 \times 10^{ - 5}$	8.95

**Figure 4:**
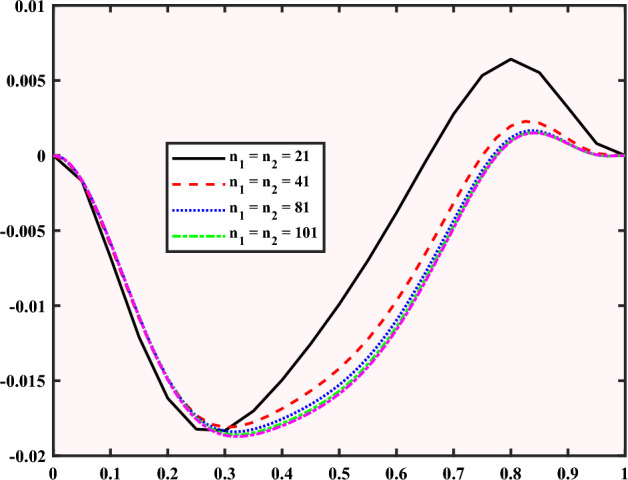
Assessment of Grid Independence for Normal Velocity Distribution along the Line y = 0.5.

Alternatively, Fig. 4 shows the convergence of our numerical results with respect to the step size, which is the distance between adjacent grid points. As the step size decreases, our numerical findings converge smoothly. This convergence confirms the stability of our numerical technique, ensuring that it does not generate spurious oscillations or divergent results. Moreover, this convergence indicates that our numerical findings are robust to changes in grid size—the total number of grid points used to discretize the domain. In other words, it implies that beyond a certain point, increasing the grid size does not significantly affect our numerical results. This reveals the reliability of our approach.

## Analysis and discussion of results

This section is devoted to presenting the analysis and discussion of the numerical results obtained in the form of tables and figures, related to the interaction of the localized magnetic field with the trihybrid nanofluid flow inside a cavity.

### Analysis of results

**Figure 5:**
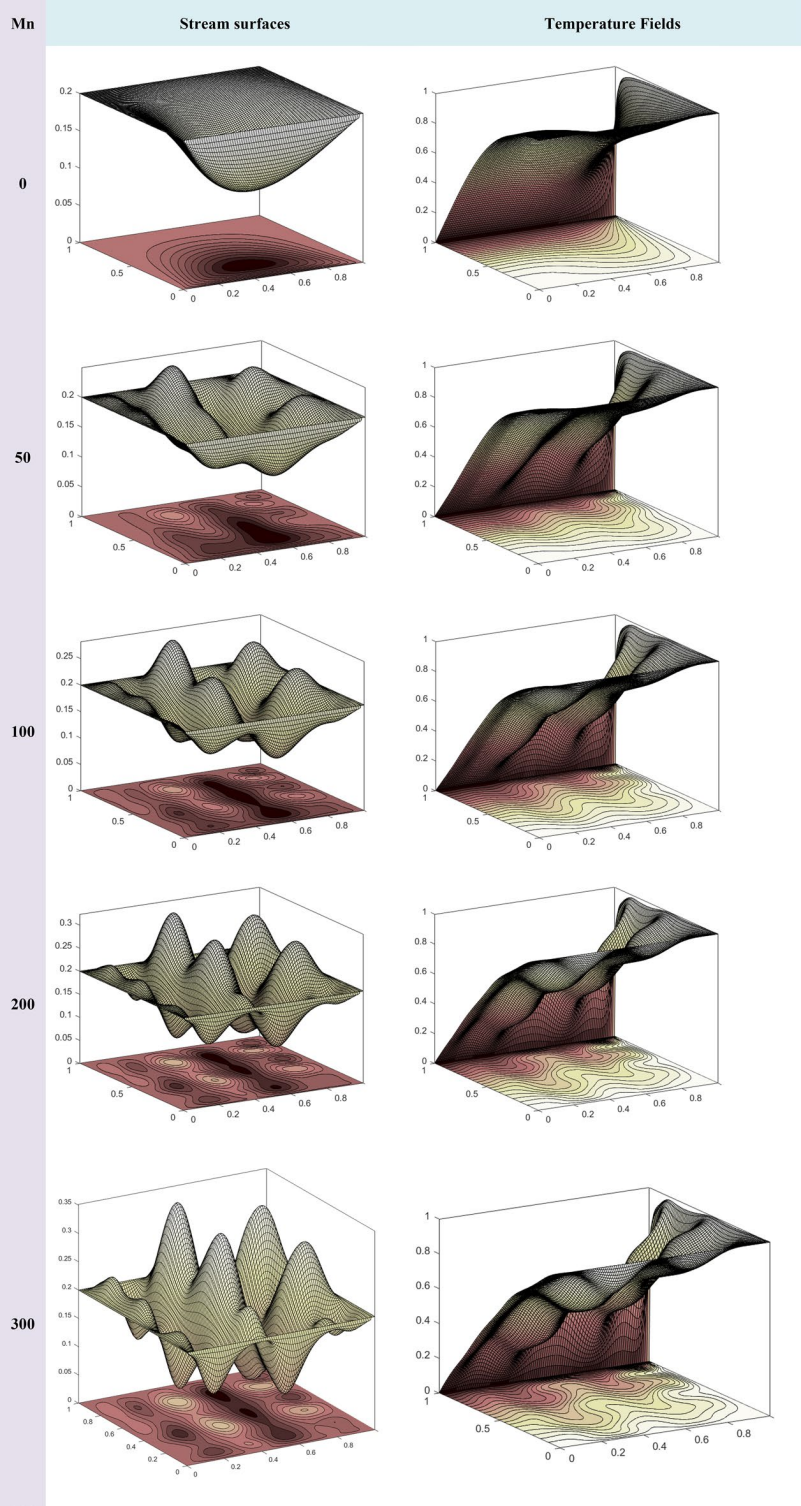
Stream surfaces and Temperature Fields for the Flow with Re = 5, L = 0, Pr = 6.2, and Different Mn.

**Figure 6:**
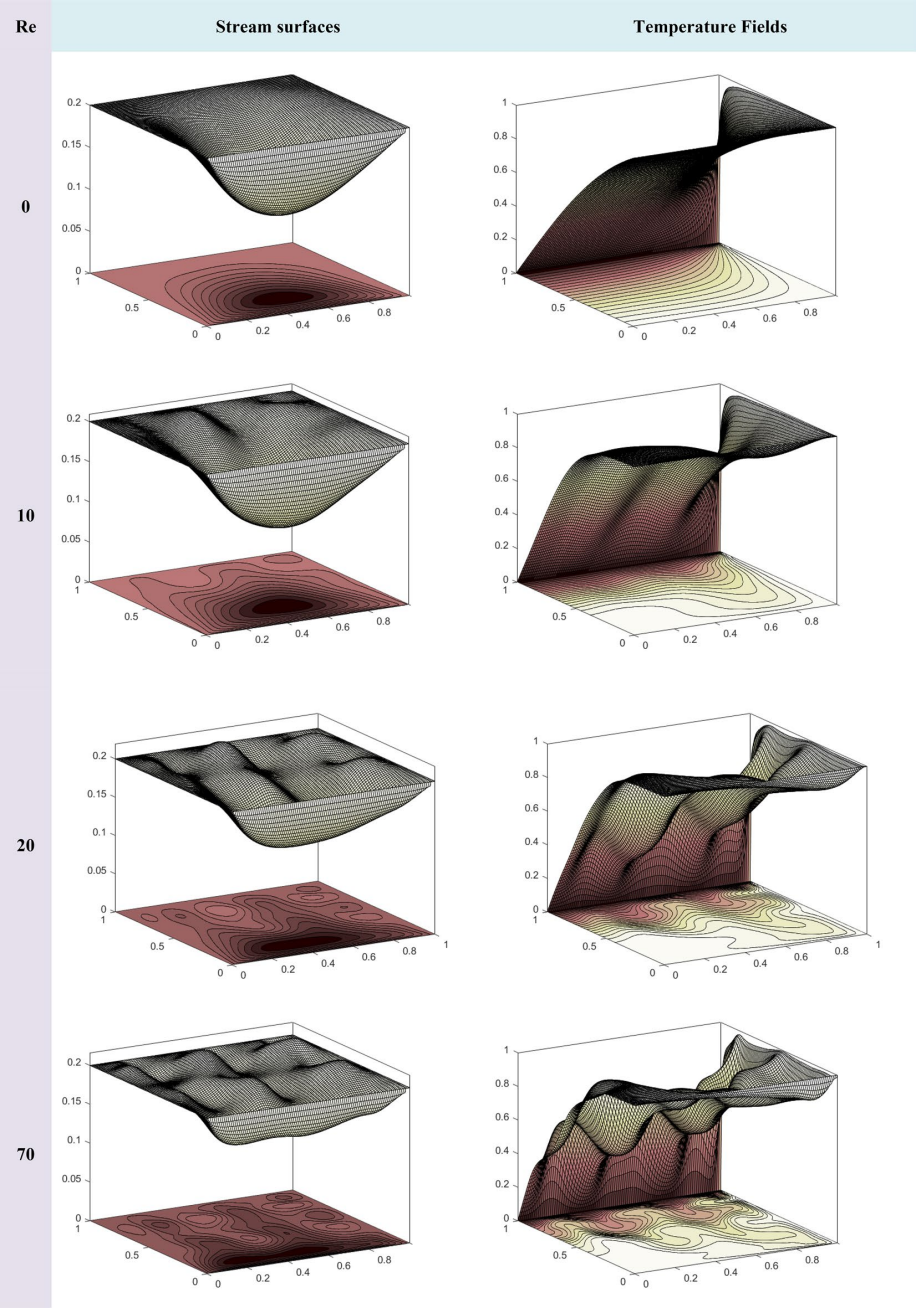
Stream surfaces and Temperature Fields for the Flow with Mn = 5, L = 0, Pr = 6.2, and Different Re.

**Figure 7:**
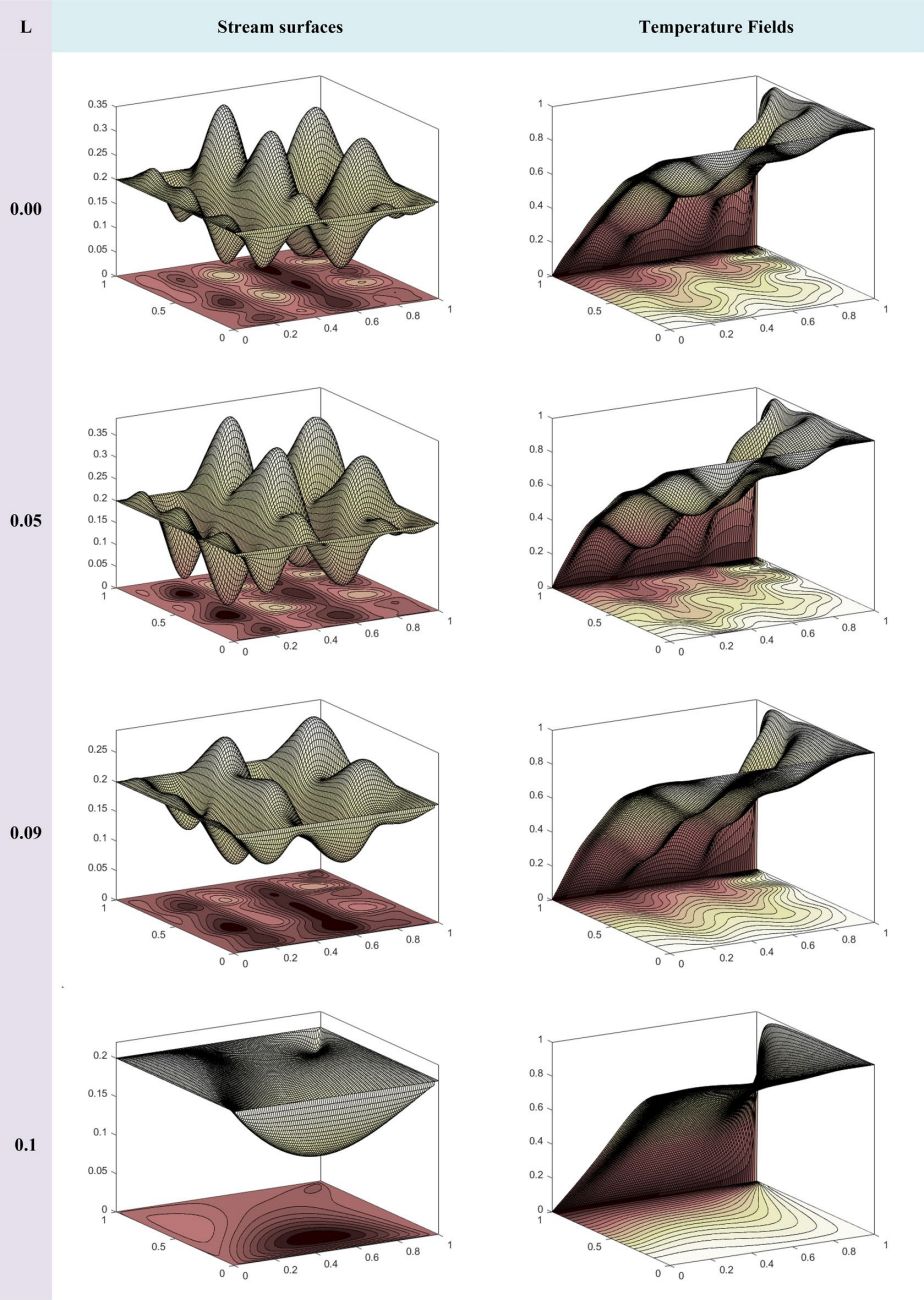
Stream surfaces and Temperature Fields for Different Values of the Magnetic Strip Length (L) for L = 0, Pr = 6.2, Mn = 150, and Re = 5.

Now, the question arises as to how the magnetic field, structured as vertical and horizontal strips, affects the main vortex within the flow. The answer may be found in Fig. 5 which reveals that the structured arrangement of vertical and horizontal magnetic strips induces notable changes in vortex dynamics, showcasing the sensitivity of the system to magnetic forces. In the absence of magnetic forces, the dominance of a single large vortex near the lower wall is evident. Upon introducing magnetic fields, the subsequent interaction with external forces and the generation of Lorentz forces extend and fragment the large vortex into smaller, varied vortices. Considering the dynamics of the present problem, a natural question of interest may be presented: How does the varying Reynolds number influence the flow pattern and the heat signature within this enclosed cavity? For this purpose, Fig. 6 is referred showing that the transition from low to high Reynolds numbers manifests in the distortion and deformation of the initially coherent vortex near the bottom walls, which is a direct consequence of the increased dominance of inertial forces relative to viscous forces. We note that the magnetic field, for the present problem, is structured as vertical and horizontal strips. In the lines to follow we try to answer how the width of these strips (where the magnetic field is localized) influences the flow dynamics and temperature distributions within the enclosure. For this, we note (from Fig. 7) that a broad magnetic field, extending across the entire flow field, has minimal impact on flow and temperature distributions, contrary to the confining of the magnetic field to specific strips which results in the formation of new vortices that disrupt the primary vortex.

The Nusselt number (*Nu*) and the product of skin friction (Cf) with the Reynolds number (Re) are given by:

$$ N_{u} = \frac{{k_{thnf} }}{{k_{nf} }}\frac{{\partial \overset{\lower0.5em\hbox{$\smash{\scriptscriptstyle\frown}$}}{\theta } }}{\partial \eta },\,\,\,\,\,{\text{CfRe}} = \frac{{2\left[ {\,\,\,\left( {1\, - \,\overset{\lower0.5em\hbox{$\smash{\scriptscriptstyle\frown}$}}{\varphi }_{1} } \right)\,\,\left( {1\, - \,\overset{\lower0.5em\hbox{$\smash{\scriptscriptstyle\frown}$}}{\varphi }_{2} } \right)\,\,\left( {1\, - \,\overset{\lower0.5em\hbox{$\smash{\scriptscriptstyle\frown}$}}{\varphi }_{3} } \right)\,\,\,} \right]^{2.5} }}{{\left[ {\left( {1 - \overset{\lower0.5em\hbox{$\smash{\scriptscriptstyle\frown}$}}{\varphi }_{3} } \right)\left\{ {\left( {1 - \overset{\lower0.5em\hbox{$\smash{\scriptscriptstyle\frown}$}}{\varphi }_{2} } \right)\left( {1 - \overset{\lower0.5em\hbox{$\smash{\scriptscriptstyle\frown}$}}{\varphi }_{1} + \overset{\lower0.5em\hbox{$\smash{\scriptscriptstyle\frown}$}}{\varphi }_{1} \frac{{\overset{\lower0.5em\hbox{$\smash{\scriptscriptstyle\frown}$}}{\rho }_{s1} }}{{\overset{\lower0.5em\hbox{$\smash{\scriptscriptstyle\frown}$}}{\rho }_{f} }}} \right) + \overset{\lower0.5em\hbox{$\smash{\scriptscriptstyle\frown}$}}{\varphi }_{2} \frac{{\overset{\lower0.5em\hbox{$\smash{\scriptscriptstyle\frown}$}}{\rho }_{s2} }}{{\overset{\lower0.5em\hbox{$\smash{\scriptscriptstyle\frown}$}}{\rho }_{f} }}} \right\} + \overset{\lower0.5em\hbox{$\smash{\scriptscriptstyle\frown}$}}{\varphi }_{3} \frac{{\overset{\lower0.5em\hbox{$\smash{\scriptscriptstyle\frown}$}}{\rho }_{s3} }}{{\overset{\lower0.5em\hbox{$\smash{\scriptscriptstyle\frown}$}}{\rho }_{f} }}} \right]}} $$

are the two relevant quantities to be discussed for the present problem. Understanding the way, the magnetic number and the Reynolds number collectively influence *CfRe* and *Nu* is another question of interest.

**Figure 8:**
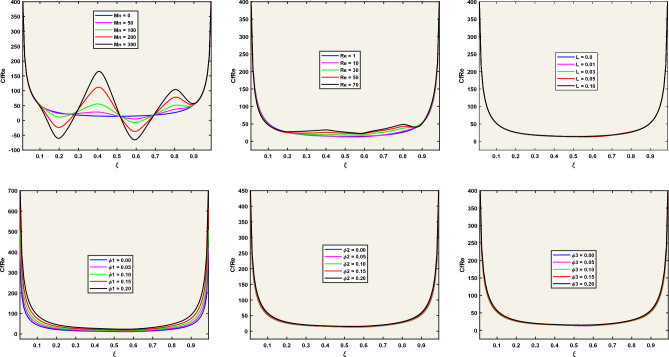
Dependence of *CfRe* on Various Factors for Fixed Mn = 5, Re = 5, and L = 0.

**Figure 9:**
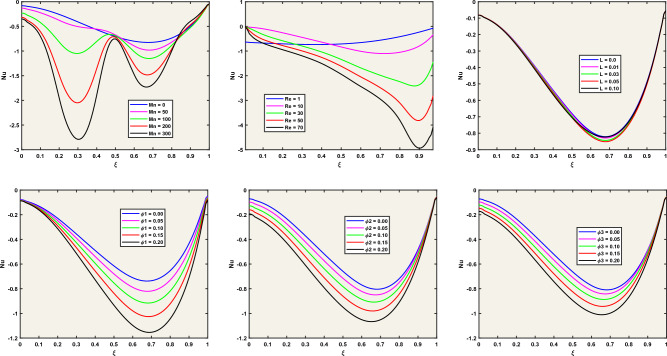
Dependence of *Nu* on Various Factors for Fixed Mn = 5, Re = 5, and *L* = 0.

Referred to Figs. 8 and 9, the magnetic parameter Mn emerges as a potent factor influencing both *Nu* and *CfRe*, while the impact of the magnetic field's width (*L*) on *CfRe* remains minimal. On the other hand, an inverse relationship between *Nu* and increasing Reynolds number is noted, with the lowest *Nu* value localized near the lower right corner of the cavity.

We note that the average Nusselt number $Nu_{av}$ and the average skin friction factor $\left( {CfRe} \right)_{av}$ along the moving lid are defined as:

$$ \begin{aligned} Nu_{av} & = \frac{{k_{thnf} }}{{k_{nf} }}\int_{\xi = 0}^{1} {\left( {\frac{{\partial \overset{\lower0.5em\hbox{$\smash{\scriptscriptstyle\frown}$}}{\theta } }}{\partial \eta }} \right)_{\eta = 0} d\xi } . \\ \left( {Cf{\text{Re}} } \right)_{av} & = \frac{{2\left[ {\,\,\,\left( {1\, - \,\overset{\lower0.5em\hbox{$\smash{\scriptscriptstyle\frown}$}}{\varphi }_{1} } \right)\,\,\left( {1\, - \,\overset{\lower0.5em\hbox{$\smash{\scriptscriptstyle\frown}$}}{\varphi } 2} \right)\,\,\left( {1\, - \,\overset{\lower0.5em\hbox{$\smash{\scriptscriptstyle\frown}$}}{\varphi }_{3} } \right)\,\,\,} \right]^{2.5} }}{{\left[ {\left( {1 - \overset{\lower0.5em\hbox{$\smash{\scriptscriptstyle\frown}$}}{\varphi }_{3} } \right)\left\{ {\left( {1 - \overset{\lower0.5em\hbox{$\smash{\scriptscriptstyle\frown}$}}{\varphi }_{2} } \right)\left( {1 - \overset{\lower0.5em\hbox{$\smash{\scriptscriptstyle\frown}$}}{\varphi }_{1} + \overset{\lower0.5em\hbox{$\smash{\scriptscriptstyle\frown}$}}{\varphi }_{1} \frac{{\overset{\lower0.5em\hbox{$\smash{\scriptscriptstyle\frown}$}}{\rho }_{s1} }}{{\overset{\lower0.5em\hbox{$\smash{\scriptscriptstyle\frown}$}}{\rho }_{f} }}} \right) + \overset{\lower0.5em\hbox{$\smash{\scriptscriptstyle\frown}$}}{\varphi }_{2} \frac{{\overset{\lower0.5em\hbox{$\smash{\scriptscriptstyle\frown}$}}{\rho }_{s2} }}{{\overset{\lower0.5em\hbox{$\smash{\scriptscriptstyle\frown}$}}{\rho }_{f} }}} \right\} + \overset{\lower0.5em\hbox{$\smash{\scriptscriptstyle\frown}$}}{\varphi }_{3} \frac{{\overset{\lower0.5em\hbox{$\smash{\scriptscriptstyle\frown}$}}{\rho }_{s3} }}{{\overset{\lower0.5em\hbox{$\smash{\scriptscriptstyle\frown}$}}{\rho }_{f} }}} \right]}}\int_{\xi = 0}^{1} {\left( {\frac{{\partial \overset{\lower0.5em\hbox{$\smash{\scriptscriptstyle\frown}$}}{u} }}{\partial \eta }} \right)_{\eta = 0} d\xi } \\ \end{aligned} $$

Now, the question arises how the magnetic field, the Reynolds number, and the nanostructure of the fluid affect the above-mentioned average quantities along the moving lid. To answer this, we refer to Tables 4, 5, 6, 7, 8 and 9 where we note substantial increases in $Nu_{av}$ and $\left( {CfRe} \right)_{av}$ with the magnetic field intensity, in contrast with the Reynolds number which primarily affects Nu only. The pronounced effects of nanoparticle type highlight the noteworthy relationships between thermal and fluid properties, emphasizing the need for careful consideration in nanofluid design.

**Table 5:** the influence of magnetic numbers on *Nu* and *CfRe* with fixed Re = 5; and Mn = 5.

${\text{Mn}}$	$\left| {{\text{Nu}}} \right|$	$\left| {{\text{CfRe}}} \right|$
0	0.5044	45.9673
50	0.5685	48.1336
100	0.7540	51.4618
200	1.0599	55.0771
300	1.2878	57.7883

**Table 6:** The Influence of Reynolds numbers on *Nu* and *CfRe* with fixed Re = 5; and Mn = 5.

${\mathbf{Re}}$	$\left| {{\text{Nu}}} \right|$	$\left| {{\text{CfRe}}} \right|$
1	0.5781	45.9742
10	0.6374	46.0433
30	1.3002	49.2697
50	1.7994	51.7839
70	2.2477	54.5683

**Table 7:** The influence of silver nanostructure on *Nu* and *CfRe* with fixed Re = 2; and Mn = 5.

$$\overset{\lower0.5em\hbox{$\smash{\scriptscriptstyle\frown}$}}{\varphi }_{1}$$(Silver)	$$\left| {{\text{Nu}}} \right|$$	$$\left| {{\text{CfRe}}} \right|$$
0.00	0.4446	38.6338
0.05	0.4922	45.8732
0.10	0.5461	54.9589
0.15	0.6077	66.5015
0.20	0.6791	81.3658

**Table 8:** The influence of alumina nanostructure on *Nu* and *CfRe* with fixed Re = 2; and Mn = 5.

$$\overset{\lower0.5em\hbox{$\smash{\scriptscriptstyle\frown}$}}{\varphi }_{2}$$(Alumina)	$$\left| {{\text{Nu}}} \right|$$	$$\left| {{\text{CfRe}}} \right|$$
0.00	0.4777	45.9607
0.05	0.5166	46.0556
0.10	0.5639	47.1380
0.15	0.6193	49.1723
0.20	0.6829	52.2189

**Table 9:** The influence of titanium dioxide nanostructure on *Nu* and *CfRe* with fixed Re = 2; and Mn = 5.

$$\overset{\lower0.5em\hbox{$\smash{\scriptscriptstyle\frown}$}}{\varphi }_{3}$$(Titanium Dioxide)	$$\left| {{\text{Nu}}} \right|$$	$$\left| {{\text{CfRe}}} \right|$$
0.00	0.4808	46.2013
0.05	0.5116	45.7407
0.10	0.5495	46.3766
0.15	0.5934	48.0141
0.20	0.6430	50.6745

Keeping in mind the importance of the magnetic field for the present problem, another question may be stated: How does increasing the width of the magnetic field (characterized by the parameter *L*) influence the average Nusselt number (*Nu*) and skin friction factor *(CfRe*)? To answer this, we refer to Table 10 where we note that an increase in the width of the strips enhances heat transfer by promoting fluid mixing, while concurrently reducing drag force for a smoother flow, thus reinforcing its potential to simultaneously enhance heat transfer and reduce drag force.

**Table 10:** The influence of localized magnetic field strips on *Nu* and *CfRe* with fixed Re = 2; and Mn = 5.

L	$\left| {Nu} \right|$	$\left| {Cf{\text{Re}} } \right|$
0.00	0.4922	45.8732
0.05	0.5085	45.9752
0.10	0.5027	45.9738
0.15	0.4732	46.2815
0.20	0.4656	46.6964

**Figure 10:**
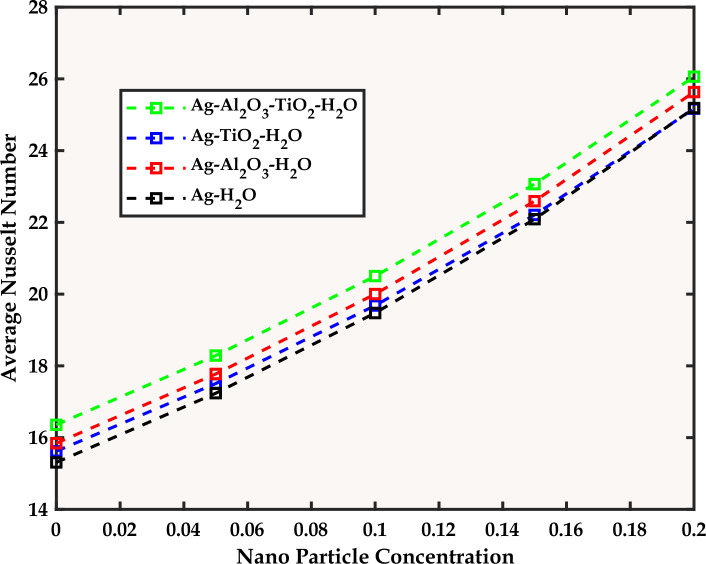
A comparative study of bi-hybrid and tri-hybrid nanofluid with fixed Mn = Re = 15.

Now we try to answer another question: How does the increasing nanoparticle volume fraction influence the average Nusselt number, and what advantages do tri-hybrid nanofluids, comprising Ag, Al_2_O_3_, and TiO_2_ nanoparticles, offer in terms of enhancing heat transfer rates? For this, we refer to Fig. 10 where we note the direct relationship between nanoparticle concentration and Nusselt number, and the superior heat transfer properties of the tri-hybrid nanofluid (with increased thermal conductivity due to the presence of different types of nanoparticles being the driving force behind elevated heat transfer rates).

**Figure 11:**
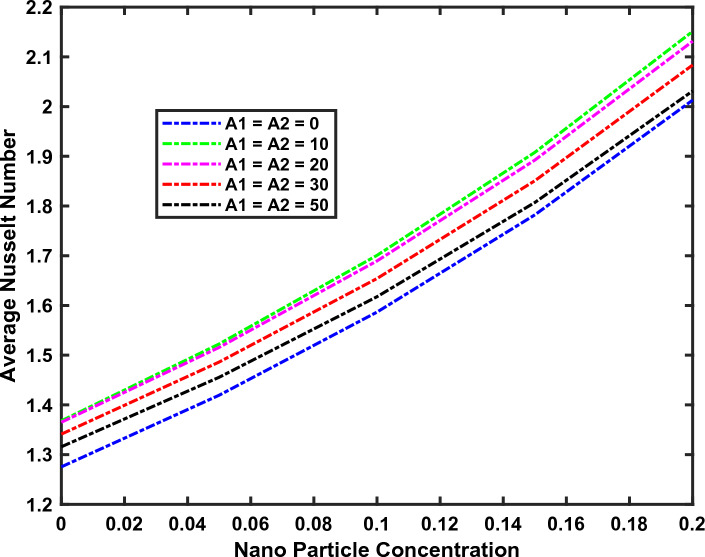
Impact of the Parameter A_1_ and A_2_ on Average *Nu* with fixed Mn = Re = 15.

**Figure 12:**
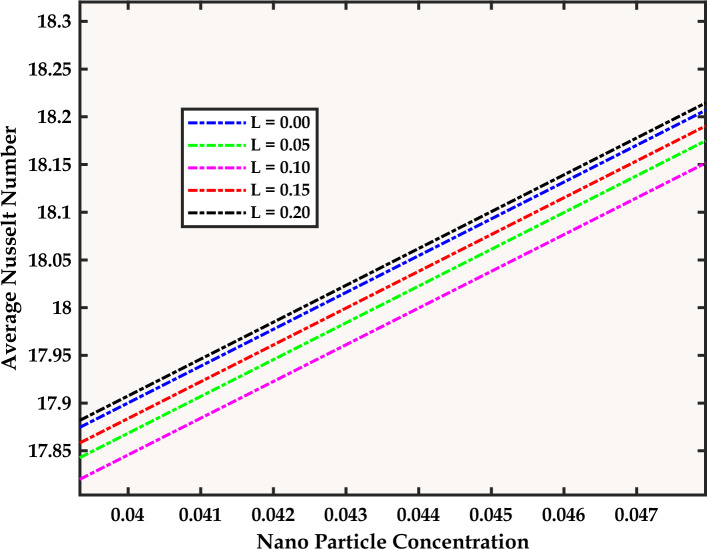
The Effect of Magnetic Strip Size on the Average *Nu* with fixed Mn = Re = 15.

Now, we analyze the way the parameters A1 and A2 (controlling the strength of the magnetic field) influence the average Nusselt number. It may be inferred from the Fig. 11 that, with the changes in A1 and A2, an increase sensitivity of *Nu* is noted, thus emphasizing the need for a more careful selection of values of these parameters. The role of the width parameter (*L*) of the magnetic field strips in influencing the Nusselt number is now analyzed. An initial decrease in Nu as *L* increases is noted (from Fig. 12) due to the inhibitory effect of the magnetic field on the flow. Subsequently, as *L* continues to rise, Nu increases which signifies a weakening of the hindering impact of the magnetic field.

### Justification and discussion of results

#### The outcomes of the magnetizing force

Figure 5 vividly illustrates the profound influence of magnetic field intensity on fluid flow within the defined regions of the cavity. The confined magnetic field orchestrates significant changes in vortex dynamics. The vortices' formation relies on the movement of the top and bottom walls, influenced by external forces. In the absence of magnetic forces, a singular large vortex dominates near the lower wall. The introduction of magnetic fields triggers the Lorentz force, generating body forces that interact with external forces, resulting in alterations to both temperature and flow fields. The magnetic sources extend the previously singular large vortex, ultimately fragmenting it into smaller and weaker vortices. These newly formed vortices exhibit diverse spin directions, reflecting the net force acting on the fluid in each region. Notably, the magnetic fields introduce marked changes to the temperature distribution within the cavity. In the absence of magnetic forces, the temperature profile remains predominantly high and smooth, with a uniform yellow color indicating the natural convection from the hot top wall to the cold bottom wall. The introduction of magnetic forces disrupts this uniformity, creating a more irregular and uneven temperature profile. This disturbance is a direct consequence of the mixing of fluid with different temperatures by the newly formed vortices, ultimately disturbing the thermal profile within the enclosure.

#### Impact of the reynolds number

Figure 6 presents the dynamic alterations in flow patterns contingent on the Reynolds number within the cavity, is referred. We note that the Reynolds number, a dimensionless metric quantifying the ratio of inertial to viscous forces in fluid flow, serves as a crucial determinant of the observed phenomena. At low Reynolds numbers (e.g., Re = 1), a singular, clockwise-spinning vortex manifests near the bottom walls, attributed to the friction between the moving walls and the fluid. As the Reynolds number increases, the vortex undergoes noticeable distortion and deformation, indicative of heightened inertial forces within the fluid. The influence of the Reynolds number extends beyond mere flow pattern alterations. Elevated Reynolds numbers amplify the significance of the magnetic term in the flow equations. This shift is a consequence of increased fluid resistance to the magnetic field under intensified inertial forces. Notably, both the Reynolds number and the magnetic parameter exhibit analogous effects on certain aspects of the flow.

#### Impact of localization

The investigation into the width of the magnetic field introduces a compelling dimension to the problem at hand. These six strips are defined as:

$$ \left. \begin{gathered} 0.1 - L < \xi < 0.2 + L{, }0.4 - L < \xi < 0.6 + L{,}\,\,0.8 - L < \xi < 0.9 + L \hfill \\ \,\,\,\,\,\,\,\,\,\,\,\,\,\,\,\,\,\,\,\,\,\,\,\,\,\,\,\,\,\,\,\,\,\,\,\,\,\,\,\,\,\,\,\,\,\,\,\,\,\,\,\,0 < \eta < 1\, \hfill \\ \end{gathered} \right\}; \left( {\text{Three Vertical Strips}} \right) $$

and

$$ \left. \begin{gathered} 0.1 - L < \eta < 0.2 + L{, }0.4 - L < \eta < 0.6 + L{, }0.8 - L < \eta < 0.9 + L{, } \hfill \\ \,\,\,\,\,\,\,\,\,\,\,\,\,\,\,\,\,\,\,\,\,\,\,\,\,\,\,\,\,\,\,\,\,\,\,\,\,\,\,\,\,\,\,\,\,\,\,\,\,\,\,0 < \xi < 1 \hfill \\ \end{gathered} \right\}; \, \left( {\text{Three Horizontal Strips}} \right) $$

The width of the strips defining the magnetic field's extent is defined by parameter *L*. A value of *L* = 0.1 indicates that the magnetic field encompasses the entire cavity, while L = 0.0 confines the magnetic field to specific strips designated as: $$\left. \begin{gathered} 0.1 < \xi < 0.2{, }0.4 < \xi < 0.6{,}\,\,0.8 < \xi < 0.9;\,\,\,\,\,0 < \eta < 1\, \hfill \\ 0.1 < \eta < 0.2{, }0.4 < \eta < 0.6{, }0.8 < \eta < 0.9;\,\,\,\,0 < \xi < 1 \hfill \\ \end{gathered} \right\}$$. The impact of this parameter *L* on flow and temperature distributions proves noteworthy. When the magnetic field spans the entire flow field, minimal effects are observed on flow and temperature distributions. However, Fig. 7 highlights significant changes when the magnetic field is confined to specific strips. This confinement instigates the formation of new vortices, disrupting the primary vortex. The isotherms, representing lines of constant temperature, adopt a zigzag trajectory due to the vigorous mixing of fluid layers at different temperatures. These findings unravel a complex relationship between magnetic field width, flow dynamics, and thermal behavior, contributing to an enriched understanding of the problem.

#### Dependency of skin-friction and nusselt number on multiple parameters

Figures 8 and 9 serve as visual representations of the *Nu* and *CfRe* profiles, respectively. The magnetic parameter (Mn) emerges as a significant influencer on both *Nu* and *CfRe*, emphasizing its crucial role in shaping heat transfer and fluid dynamics. Surprisingly, the width (*L*) of the magnetic field strip within the flow field exhibits a negligible effect on *CfRe*. The Nusselt number (*Nu*) exhibits a substantial decrease with increasing Reynolds numbers (Re), reaching its lowest value near the lower right corner of the cavity, particularly at higher Re values. On the other hand, *CfRe*, dependent on various factors, shows minor differences among them. Nanoparticles in the fluid, including Silver, Aluminium oxide, and Titanium dioxide, have a negligible impact on *CfRe*, compared to *Nu*. An interesting observation emerges regarding the magnetic field's impact on *Nu* throughout the flow field. Contrary to expectations, the magnetic field doesn't consistently increase or decrease Nu. This noteworthy observation regarding the inconsistent impact of the magnetic field on *Nu* may be a potential research direction in search of optimal magnetic strip width.

#### Effect for the solid volume concentration (Ag, Al_2_O_3_, TiO_2_)

Table 5 underscores the substantial impact of increasing magnetic field intensity (Mn), leading to a remarkable elevation in both *Nu* and *CfRe*. The maximum increases are noteworthy, reaching 155% for *Nu* and 26% for *CfRe*. Conversely, Table 6 reveals that changing the Reynolds number (Re) primarily affects *Nu*, with a minor influence on *CfRe*. Higher Re values correspond to increased *Nu* and *CfRe*, emphasizing the divergent roles of Mn and Re for the two physical quantities. The impact of Mn on the flow, amplifying drag force and reducing flow rate, contrasts with that of Re on heat transfer. The numerical results presented in Tables 4 and 5 affirm the pivotal roles played by the magnetic field and Reynolds number in shaping heat transfer and flow dynamics within the cavity. These findings align with previous notable works, such as Animasaun et al.^49–52^, confirming the consistency and significance of the observed trends.

Tables 7, 8 and 9 delve into the effects of different nanoparticle types on the heat transfer rate of the nanofluid. The type of nanoparticle emerges as a critical determinant, with silver (Ag) nanoparticles exhibiting the most pronounced increase in both *Nu* and *CfRe*. This underscores the intricate interplay between thermal and fluid properties in Ag nanofluids. In contrast, alumina (Al_2_O_3_) and titanium dioxide (TiO_2_) nanoparticles show comparatively weaker relationships, suggesting potential trade-offs between thermal and fluid properties. These results underscore the importance of choice of the nanoparticle type in the design of nanofluids for heat transfer applications, which are consistent with previous notable works by Animasaun et al.^16,53^.

Table 10 provides valuable details about the effects of increasing the width of the magnetic field (parameter *L*) on the average Nusselt number ($Nu_{av}$) and average skin friction factor $\left( {CfRe} \right)_{av}$. We note that a slight decrease in Nu accompanies a slight increase in $\left( {CfRe} \right)_{av}$ as the magnetic field width expands. This phenomenon is due to the enhancing effects of the magnetic field on fluid mixing, subsequently improving heat transfer and reducing drag force.

The increase in *Nu* is explained by the force exerted by the magnetic field, deflecting the fluid flow and promoting more effective mixing. This mixing facilitates the convergence of hot and cold fluid layers, augmenting heat transfer by bringing them closer to each other. Simultaneously, the decrease in $\left( {CfRe} \right)_{av}$ results from the opposing force generated by the magnetic field, diminishing the drag force on the fluid. This allows for a smoother flow, thereby reducing friction between the fluid and the cavity walls and resulting in a lowering of $\left( {CfRe} \right)_{av}$. The noteworthy observation suggests that extending the coverage of the magnetic field is beneficial, as it not only improves heat transfer by enhancing fluid mixing but also reduces drag force, contributing to a smoother flow within the cavity.

#### Thermal characteristics of the nanostructures: a comparison

Referred to Fig. 10, a notable and nearly linear increase in the average Nusselt number $Nu_{av}$ is observed with the rise in nanoparticle volume fraction. This trend indicates that an augmented nanoparticle concentration enhances the heat transfer rate within the fluid. Furthermore, the tri-hybrid nanofluid, consisting of Ag, Al_2_O_3_, and TiO_2_ nanoparticles, outperforms the conventional fluid in significantly increasing the average Nusselt number. The enhanced Nu with increased nanoparticle concentration is attributed to the increased thermal conductivity of the fluid induced by the nanoparticles. This increased thermal conductivity allows the nanoparticles to carry more heat, thereby amplifying the overall heat transfer rate. The synergistic collaboration of Ag, Al_2_O_3_, and TiO_2_ nanoparticles in the tri-hybrid nanofluid contributes to superior thermal conductivity. Specifically, Ag's excellent heat conductivity, coupled with Al_2_O_3_ and TiO_2_'s heat absorption capabilities, results in a fluid with superior heat transfer properties. Thus, it may be suggested that tri-hybrid nanofluids hold significant promise for enhancing heat transfer in various applications, including heat exchangers, solar collectors, electronic cooling, and medical applications.

#### The impact of the magnetic field intensity on the flow and heat transfer

In Fig. 11, the influence of the parameters A1 and A2 on the average Nusselt number (*Nu*) is revealed. These parameters dictate the strength of the magnetic field, and the observed sensitivity of Nu to changes in A1 and A2 is noteworthy. Notably, Nu exhibits a higher sensitivity when these parameters are smaller. The examination of numerical results indicates that variations in A1 and A2, especially when smaller, have a more pronounced impact on *Nu*. This heightened sensitivity suggests that fine-tuning the strength of the magnetic field through adjustments in A1 and A2 can significantly affect the heat transfer characteristics within the system. The tuning of these parameters is thus important for optimizing the magnetic field strength for enhanced heat transfer. We have also noted that beyond A1 = A2 = 50, no significant change occurs in the numerical results, thus making it a suitable choice for the numerical computations throughout this paper.

#### The impact of the magnetic field localization

The role the width parameter (*L*) of the magnetic field strips plays in influencing the Nusselt number is now discussed. The magnetic field is assumed in the form of horizontal and vertical positioned strips each having a variable width being controlled by the parameter *L*, where *L* = 0.2 indicates that the magnetic field spans the entire cavity, and *L* = 0.0 confines the magnetic field to the designated strips. Figure 12 illustrates the variations in the Nusselt number (*Nu*) concerning the changing parameter *L*. As *L* increases, the figure reveals a distinctive pattern in the behavior of *Nu*. Initially, there is a decrease in Nu, indicating that the magnetic field created by the strips impedes the fluid flow. However, as the width of the strips continues to increase, there is a subsequent rise in *Nu*. This phenomenon suggests that, with the expanding width of the magnetic strips, the inhibiting effect of the magnetic field diminishes, allowing for improved fluid flow.

## Concluding remarks

The idea of this research is to investigate the numerical simulation of a tri-hybrid nanofluid flow inside a lid-driven cavity under the influence of a magnetic field (formed through a vertical strip) by using a single-phase model to characterize the tri-hybrid nanofluid consisting of Silver (Ag), Aluminium Oxide (Al_2_O_3_), and Titanium dioxide (TiO_2_). Based on the results of this study, the following main findings can be summarized:

- In the absence of a magnetic field, a single vortex forms near the lower lids of the enclosure. When magnetic sources are introduced, they initially strengthen the primary vortex in the upper part of the cavity, followed by the appearance of some smaller and less robust vortices in the lower region. These secondary vortices exhibit clockwise or counterclockwise rotation.
- The flow experiences varying influences from the magnetic fields generated by the vertical and horizontal strips. These fields interact with external forces, resulting in the generation of additional vortices that rotate in diverse directions.
- In the absence of magnetic forces, the flow field exhibits predominantly smooth and yellowish-colored patterns, indicative of elevated temperatures. Isotherms also display increased density near the top and bottom walls. However, the introduction of magnetic forces disrupts any linear temperature distribution within the cavity. The flow engenders new vortices that mix fluids with differing temperatures, thus disturbing the thermal patterns within the enclosure.
- A magnetic field encompassing the entire flow field has a relatively modest impact on flow and temperature distributions. In contrast, localized magnetic field intensity generates fresh vortices while eliminating primary ones.
- The nanoparticulate nature of the fluid has minimal influence on skin friction. The Nusselt number (*Nu*) is more significantly affected by parameters such as thermal conductivity than by factors like viscosity and density, owing to the distinctive physical attributes of the nanoparticles.
- The localized magnetic field does not consistently alter the Nusselt number (*Nu*). There may exist an optimal width for the magnetic pathway to maximize *Nu*.
- Increased magnetic field strength enhances both the skin friction factor (*CfRe*) and Nu, with improvements of up to 26% and 155%, respectively. The Reynolds number significantly impacts Nu but has a lesser effect on *CfRe*.
- Nanoparticles enhance both *Nu* and *CfRe*, albeit with variations depending on the nanoparticle type. Silver (Ag) exhibits the most substantial increase in both parameters (52% and 110%), underscoring a robust connection between the nanofluid's thermal and fluid properties. In contrast, Alumina (Al_2_O_3_) and Titanium Dioxide (TiO_2_) nanoparticles demonstrate lower increases in both *Nu* (43% and 34%) and *CfRe* (14% and 10%), suggesting a weaker correlation with the nanofluid's characteristics.
- Tri-hybrid nanofluids composed of Ag, Al_2_O_3_, and TiO_2_ nanoparticles exhibit greater enhancements in average Nu compared to simple nanofluids containing only Ag, Al_2_O_3_, or TiO_2_ particles.
- *Nu* varies with the strip width (*L*), initially decreasing and then increasing as the strip widens. Its response is more pronounced with higher nanoparticle concentrations when $$\overset{\lower0.5em\hbox{$\smash{\scriptscriptstyle\frown}$}}{\varphi }_{1}$$, $$\overset{\lower0.5em\hbox{$\smash{\scriptscriptstyle\frown}$}}{\varphi }_{2}$$ and $$\overset{\lower0.5em\hbox{$\smash{\scriptscriptstyle\frown}$}}{\varphi }_{3}$$ and are elevated.

## Data Availability

The datasets generated during the current study are available from the corresponding author upon reasonable request.
